# Heterogeneous patterns of heterozygosity loss in isolated populations of the threatened eastern barred bandicoot (*Perameles gunnii*)

**DOI:** 10.1111/mec.17224

**Published:** 2023-11-28

**Authors:** John G. Black, Anthony R. J. van Rooyen, Dean Heinze, Robbie Gaffney, Ary A. Hoffmann, Thomas L. Schmidt, Andrew R. Weeks

**Affiliations:** ^1^ School of Biosciences The University of Melbourne Melbourne Victoria Australia; ^2^ Cesar Australia Brunswick Victoria Australia; ^3^ Research Centre of Applied Alpine Ecology La Trobe University Melbourne Victoria Australia; ^4^ Department of Natural Resources and Environment Hobart Tasmania Australia

**Keywords:** conservation genomics, genetic diversity, isolation, marsupial, population genetics

## Abstract

Identifying and analysing isolated populations is critical for conservation. Isolation can make populations vulnerable to local extinction due to increased genetic drift and inbreeding, both of which should leave imprints of decreased genome‐wide heterozygosity. While decreases in heterozygosity among populations are frequently investigated, fewer studies have analysed how heterozygosity varies among individuals, including whether heterozygosity varies geographically along lines of discrete population structure or with continuous patterns analogous to isolation by distance. Here we explore geographical patterns of differentiation and individual heterozygosity in the threatened eastern barred bandicoot (*Perameles gunnii*) in Tasmania, Australia, using genomic data from 85 samples collected between 2008 and 2011. Our analyses identified two isolated demes undergoing significant genetic drift, and several areas of fine‐scale differentiation across Tasmania. We observed discrete genetic structures across geographical barriers and continuous patterns of isolation by distance, with little evidence of recent or historical migration. Using a recently developed analytical pipeline for estimating autosomal heterozygosity, we found individual heterozygosities varied within demes by up to a factor of two, and demes with low‐heterozygosity individuals also still contained those with high heterozygosity. Spatial interpolation of heterozygosity scores clarified these patterns and identified the isolated Tasman Peninsula as a location where low‐heterozygosity individuals were more common than elsewhere. Our results provide novel insights into the relationship between isolation‐driven genetic structure and local heterozygosity patterns. These may help improve translocation efforts, by identifying populations in need of assistance, and by providing an individualised metric for identifying source animals for translocation.

## 1 INTRODUCTION

Small, isolated populations are at an increased risk of inbreeding and can experience strong genetic drift (Charlesworth & Willis, 2009; Lynch et al., 2016; Wright, 1931, 1932). Both processes cause small populations to be more homozygous over time, either by fixing alleles and thus removing heterozygosity from the population (Lynch et al., 2016) or by increasing the likelihood that genomic regions inherited from each parent will be identical (Charlesworth & Willis, 2009). Identical genomic regions can result in the expression of recessive, deleterious alleles causing a reduction in fitness known as inbreeding depression, while reduced genetic diversity causes populations to be less adaptable to environmental change and increases their vulnerability to disease and pathogens (Hoffmann et al., 2017; Miller et al., 2011; Stewart et al., 2017; Weeks et al., 2016; Wright, 1931). Populations then risk being caught in a feedback loop where increased mortality and reduced breeding success caused by low heterozygosity in turn work to keep heterozygosity and population size low, which can result in local extirpation (Hoffmann et al., 2017; Weeks et al., 2013, 2016, 2017).

In small populations, mutation rates are insufficient to generate new variation, while in isolated populations no new genetic variation can be imported, making population size and isolation key risk factors for genetic decline (Hoffmann et al., 2017; Spielman et al., 2004). Fragmentation also has a deleterious effect on genetic variation in populations due to the loss of genetic exchange (Fletcher et al., 2018; Gilbert‐Norton et al., 2010; Haddad et al., 2015). If migration is low and a small population cannot accumulate mutations at a sufficient rate to counteract genetic drift, fragmented populations will become increasingly homozygous and inbred (Haddad et al., 2015), resulting in an increased genetic load that can affect population fitness and adaptive potential (Hoffmann et al., 2021). This makes identifying isolated populations and estimating rates of migration and genetic drift of key concern for conservation managers (Frankham, 1996; Ralls et al., 2020; Ramalho et al., 2018).

Although it is individuals that experience the deleterious effects of low heterozygosity, most genomic studies report heterozygosity at a population level. This may in part be because bioinformatic pipelines that are optimised for assessing genetic structure among populations are poorly suited for assessing heterozygosity among individuals (Schmidt et al., 2023), and thus multiple workflows are required to assess both. Genomic studies assessing heterozygosity effects on individual fitness have found strong associations whether heterozygosity is assessed through the average frequency of heterozygous sites across autosomes (Scott et al., 2020), also known as autosomal heterozygosity (Schmidt et al., 2021), or through runs of homozygosity (Ceballos et al., 2018), and these two measures can be highly correlated (Kardos et al., 2018, 2021; Mathur et al., 2023). Heterozygosity often decreases before a threatened species has undergone significant population contractions (Spielman et al., 2004), although it is worth noting that scenarios such as recent bottlenecks may threaten populations without immediately impacting heterozygosity. Assessing heterozygosity at the individual level can also reveal how heterozygosity is structured within populations, which may show spatial or temporal variation or may exhibit unusual distributions. If heterozygosity has declined in a wild population, individual heterozygosity estimates can be used to investigate the geographical and temporal structure of these declines. If there are neighbourhoods of individuals with low heterozygosity in a population, this indicates a failure of these individuals to mate with less genetically similar individuals, and results in local inbreeding. Low individual heterozygosity may therefore help indicate the early warning signs of a local collapse, which could then have repercussions on the rest of the population.

Individual heterozygosity estimates also help evade biases in parameter estimates caused by unrecognised spatial or temporal structure in a population sample. If wild populations are not well mixed, which often occurs if neighbourhood size (Wright, 1946) is small (<100) or when related individuals do not disperse far from parents, animals at population boundaries may have lower heterozygosity than animals from population cores (Battey et al., 2020). This can yield deceptive results if sampling fails to account or test for spatial arrangement when parameters are reported at the population level (Chikhi et al., 2010; Mazet et al., 2016; Städler et al., 2009). Furthermore, populations that are not well mixed will also experience non‐random breeding, which causes different areas within a population to exhibit different pools of homozygosity (Battey et al., 2020). This causes Wright's inbreeding coefficient (*F*_IS_) to appear elevated due to a perceived excess of homozygous sites, a continuous‐space analogue of a Wahlund effect. If *F*_IS_ is interpreted as a proxy for the degree of inbreeding or outbreeding present, it can lead to erroneous conclusions when sampling regimes are uneven (Schmidt et al., 2023). Furthermore, *F*_IS_ only describes inbreeding in the analysed samples, and therefore, if not temporally replicated, only describes inbreeding in a single generation. These factors are critical for interpreting genetic parameters in studies of low‐density or cryptic species (such as endangered species) where sampling is typically patchy.

In this paper, we use genome‐wide sequence data to assess population structure, gene flow and individual heterozygosity in wild populations of the eastern barred bandicoot (*Perameles gunnii*, Gray 1838) from Tasmania, Australia. This represents the first genomic investigation of this species across its wild range. Previous work has characterised Tasmanian *P. gunnii* as being genetically divided into northern and southern groups (Robinson et al., 1993; Weeks et al., 2013), which follows the likely extirpation of *P. gunnii* in central Tasmania. Here, we describe additional discrete and continuous genetic structure among seven demes in north and south Tasmania, and report low levels of gene flow between them. We show how individual autosomal heterozygosities exhibit considerable variation within demes and across space, but nevertheless, identify specific areas where individuals with low heterozygosity are common. Understanding how heterozygosity is distributed within and between populations is likely to be important information for conservation managers seeking to use population mixing as an early intervention to arrest genetic declines in vulnerable populations. In particular, this may help avoid the severe declines and subsequent population bottleneck experienced by *P. gunnii* on mainland Australia and the intensive genetic management required for its persistence (Weeks et al., 2013).

## 2 METHODS

### 2.1 Study species

*Perameles gunnii* is a terrestrial marsupial native to the southern and eastern parts of Australia. On mainland Australia, the species cannot persist outside of terrestrial predator‐proof fenced havens or islands due to the widespread presence of red foxes (*Vulpes vulpes*, Linnaeus 1758) (Radford et al., 2018). Foxes in Tasmania have failed to establish a stable population, despite incursions, allowing *P. gunnii* to persist in the wild (Caley et al., 2015). *Perameles gunnii* is highly impacted by roads and predation by feral cats (*Felis catus*, Linnaeus 1758) (Dufty, 1994a), and even appears to be sensitive to the presence of non‐predator exotic species (Randall et al., 2023). Populations of *P. gunnii* are also declining due to environmental change (resulting in reduced rainfall) and the conversion of land for agriculture (resulting in habitat loss and fragmentation and patch isolation), the latter of which has likely seen the local extirpation of this species from midland areas of Tasmania since 1989 or earlier (Driessen et al., 1996; Mallick et al., 1998; Ramalho et al., 2018; Robinson et al., 1991; Weeks et al., 2013).

Female adults typically produce up to three litters of 2–3 offspring each year, and offspring mature at 4–6 months old, but reproduction is often depressed in summer and during times of drought (Winnard & Coulson, 2008). Few studies have looked at dispersal in *P. gunnii*, but in other peramelids juveniles disperse approximately 1 km on average from the home range of their maternal parent, based on observations of spatial autocorrelation (*Perameles nasuta*; Piggott et al., 2018) and radio‐tracking (*Isoodon obesulus*; Robinson et al., 2018). Post‐dispersal, *P. gunnii* establish a home range of 2–4 hectares, with male and female territories overlapping but exclusive to individuals of the same sex (Mallick et al., 2000). At two predator‐proofed havens of 450+ hectares, *P. gunnii* individuals are frequently recaptured in their established territory for several years (Black, 2019), suggesting that dispersal rates after establishment and neighbourhood size are both low.

Tasmanian *P. gunnii* and those from the Australian mainland are sometimes considered as separate (albeit undesignated) subspecies (Department of the Environment, Water, Heritage and the Arts, 2008), but there is little genetic evidence for this (Weeks et al., 2013). Populations have been separated since the flooding of the last land bridge (the Bassian Plain) approximately 14,000 years ago (Lambeck & Chappell, 2001), and some genetic differentiation has been observed using mitochondrial DNA (Robinson, 1995), nuclear variable number tandem repeat (VNTR) loci (Robinson et al., 1993) and 10 microsatellite loci and two mitochondrial genes (Weeks et al., 2013). While Tasmanian animals are often anecdotally reported as larger, no research has been conducted to test whether this has a genetic underpinning or represents phenotypic plasticity to a cooler climate (e.g. Bergmann's rule). The two subspecies exhibit similar habitat requirements, feeding behaviours and dispersal characteristics (Dufty, 1994b; Mallick et al., 2000; Winnard & Coulson, 2008).

### 2.2 Genetic sampling, DNA extraction and genotyping

**FIGURE 1:**
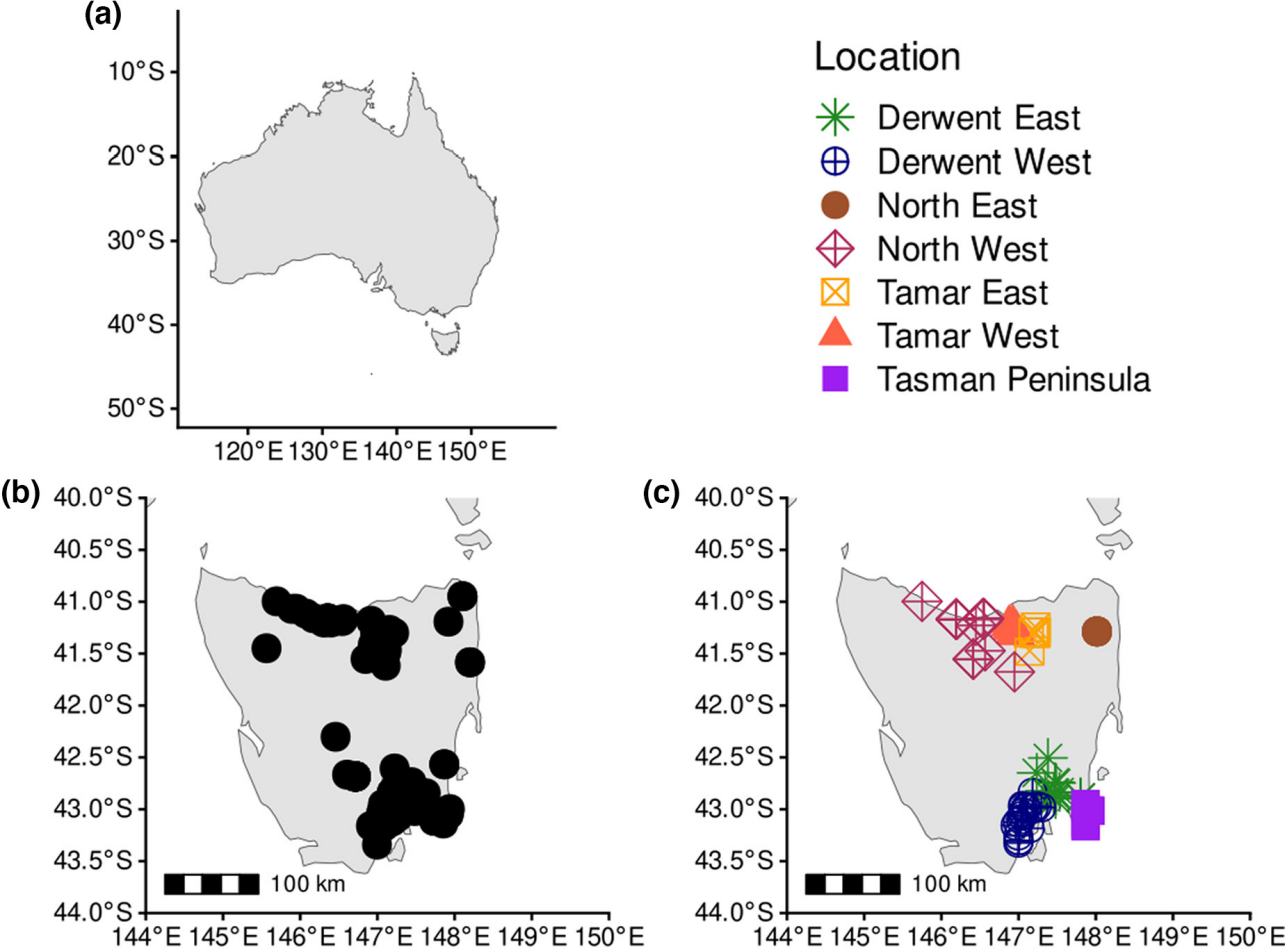
Maps of Australia (a, for reference), and Tasmania, overlaid with post‐1990 species distribution (b) and sample locations with a priori group assignments for 85 Tasmanian *Perameles gunnii* samples (c), on a Goode projection map. Species distribution based on post‐1990 sighting data is available at the Atlas of Living Australia, a modelled distribution based on species habitat is available from the Australian Government Species Profile and Threats Database for this species (Department of Climate Change, Energy, Environment and Water, 2023).

Tissue samples were provided by the Department of Primary Industries, Parks, Water and Environment (DPIPWE) Tasmania for genetic analysis for a previous study (Weeks et al., 2013) under AEC 05186. DPIPWE obtained tissue from 117 Tasmanian *P. gunnii*, dated 2008–2011, through wild trapping and roadkill collection. Tissue samples were stored at −20°C from 2011 until 2022, when DNA was extracted from tissue samples using a Qiagen DNEasy Blood and Tissue kit according to manufacturer's protocol, and extracted DNA was then stored at −20°C. Limited precise coordinate data were available for Tasmanian samples; for samples that did not have coordinates the nearest settlement had instead been recorded, resulting in a location accurate to approximately 5 km for all samples. Samples did not have metadata distinguishing which were roadkill and which were live‐trapped. Samples were a priori assigned to seven populations (hence: ‘demes’) for analysis, based on known geographical barriers, and broadly grouped into four northern and three southern demes (Figure 1). The northern demes are as follows: the North West, Tamar West, Tamar East and North East, while the southern demes are as follows: Derwent West, Derwent East and Tasman Peninsula. The multiple Tamar and Derwent demes are separated by the Tamar and Derwent Rivers, respectively, while the Tasman Peninsula is separated from Derwent East by the 7 m wide, 120‐year‐old Denison Canal. Herein, we assess which of these demes are genetically isolated, whether our a priori groupings are valid, and whether structure between demes is discrete or continuous. Genome‐wide sequence data were generated by DArTSeq (Cruz et al., 2013; Kilian et al., 2012), with raw data produced in .fastq format.

### 2.3 Genotype and sample filtering

Fastq files were demultiplexed using the process_radtags command from the Stacks2 v2.64 suite (Catchen et al., 2013; Rochette et al., 2019), with the additional settings ‘‐‐renz‐1 pstI ‐c ‐q ‐r ‐s 20’ (restriction enzyme cut site pstI, a minimum rolling PHRED score of 20) to discard low‐quality reads. 5′ adapters and Illumina universal sequencing adapters were removed using cutadapt v4.4 (Martin, 2011), leaving sequences of variable length that were aligned to our draft genome scaffold using BWA‐MEM v0.7.12 (Li, 2013). FASTA v1.0.3 (Blankenberg, et al., 2014) summary statistics for this genome were produced on the Galaxy Australia portal and are available in Table S1; briefly, it is a scaffolded assembly produced using PacBio and Illumina data, sequenced from an animal from Victoria, mainland Australia. DArTseq alignments to this genome were sorted and indexed using the samtools v1.16 commands ‘view’, ‘sort’ and ‘index’ sequentially (Li et al., 2009). Finally, samples were catalogued using gstacks from the Stacks 2 suite. For downstream filtering, .bam files were processed using populations (Stacks2).

We first examined samples for excessive missing data, using the populations filter ‘‐R 0.7’ to set per‐locus missingness (SNP completeness) across demes at a call‐rate of >70%, and calculated per‐sample missingness with vcftools v1.16 (Danecek et al., 2011). We selected a threshold of <10% missing data per sample for inclusion, with 85 samples (73%) retained at this threshold. A threshold of <30% missing data would have resulted in 96 samples (82%) retained, but with fourfold fewer SNPs. The program gstacks was rerun to form a new catalogue containing the 85 retained samples. ‘Populations’ was rerun using the following parameters: an increased per‐locus call‐rate threshold of >85% across demes (‐R 0.85), a per‐locus call‐rate of >50% within all seven demes (‐r 0.5, ‐p 7) and a minimum minor allele count of 3 (‐mac 3). For downstream analyses, loci were further filtered at a minimum depth of 5X and maximum depth of 95X using vcftools (‐‐min‐meanDP 5, ‐‐max‐meanDP 95), as determined by Li's maximum read depth (Li, 2014), retaining 4282 SNPs. For analysis in Structure (Porras‐Hurtado et al., 2013) and TreeMix (Pickrell & Pritchard, 2012), removal of linkage is essential, therefore SNPs were thinned to 10,000 bp distances with vcftools (‐‐thin 10,000), retaining 3020 SNPs for these analyses.

We assessed samples for full‐sibling, half‐sibling, or parent‐offspring relationships using the function ‘pcrelate’ from the R v4.2.3 package GENESIS v2.26.0 (Gogarten et al., 2019; R Core Team, 2021), to ensure that our subsequent analyses were not biased by high levels of identity by descent. For each dyad, PCRelate reports kinship coefficients (*K*) and probability of sharing no alleles identical by descent (*K*_0_). *K*_0_ distinguishes parent‐offspring pairs, which have *K*_0_ approaching 0, from full‐ and half‐ siblings which will have *K*_0_ of 0.25 or higher. We observed no parent‐offspring pairs and six possible half‐ or full‐sibling pairs (Figure S1a) out of 3570 possible pairs, distributed across 4 of our demes with no more than 2 per deme. As this distribution was well within the acceptable threshold for kin sampling of Waples and Anderson (2017), no samples were excluded on this basis. Sex data were available for only 49 out of 85 samples (22 female:27 male); however, this is still sufficient to identify SNPs exhibiting sex‐biased heterozygosity. We used the function ‘gl.report.sexlinked’ from the package dartR v2.9.7 (Gruber et al., 2018; Mijangos et al., 2022) and the unpackaged function ‘filter.sex.linked’ (Robledo‐Ruiz et al., 2023) to examine loci for sex‐based differences in heterozygosity and found zero loci that displayed sex chromosome‐linked heterozygosity patterns in our panel of 4282 SNPs (Figure S1b).

### 2.4 Genetic structure analysis

To visualise genetic structure among individuals, we generated a principal component analysis (PCA) of pairwise genetic differences in RStudio v2022.07.2 (RStudio Team, 2020) using the packages stats v4.3.1 (R Core Team, 2021) and ggplot2 v3.4.2 (Wickham, 2016). We used Structure v2.3.4 (Porras‐Hurtado et al., 2013) to assess genetic clustering of individuals. Ten replicate runs were created for each value of *K* from 2 to 8, each with a random seed, a burn‐in of 50,000 iterations, and analysis of 250,000 iterations. Graphs from the 10 runs at each value of *K* were aggregated using CLUMPAK v1.1 (Kopelman et al., 2015) and the probable number of ancestral clusters was selected based on Δ*K* from Structure Harvester v0.6.94 (Earl & vonHoldt, 2012; Evanno et al., 2005). The programs fineRADstructure v0.3.1 and Radpainter v0.3.1 were used to estimate recent shared coancestry between individuals by creating a heatmap and dendrogram of shared RADtag microhaplotypes (Malinsky et al., 2018). PCA, Structure and fineRADstructure all operate without utilising a priori information about the seven demes, and these would therefore serve as a test of these hypothetical populations.

Isolation by distance was assessed within demes, within northern and southern regions, and across Tasmania using Mantel tests of logarithmic pairwise geographic distance and Rousset's â (Rousset, 2000), a metric of individual pairwise genetic distance, calculated in SPAGeDi v1.5 (Hardy & Vekemans, 2002). Mantel tests were run using the R package ade4 v1.7 (Dray & Dufour, 2007; Thioulouse et al., 2018). We used ConStruct v1.0.5 (Bradburd et al., 2018) to assess patterns of discrete and continuous genetic clustering. We ran two models in ConStruct, one with only discrete structure (analogous to Structure), the other accounting for the decay of genotype similarity across continuous space. We also used spatial principal components analysis (sPCA) run in adegenet v2.1.10 (Jombart, 2008; Jombart & Ahmed, 2011; Jombart et al., 2008) to assess global and local structures of genetic differentiation and to spatially map differentiation clines. To assess genetic differentiation between demes, we estimated genetic distance (*F*_ST_) using the packages vcfR v1.14.0 (Knaus & Grünwald, 2017) StAMPP v1.6.3 (Pembleton et al., 2013), and assessed statistical significance by bootstrapping (10,000 bootstraps) with a Bonferroni correction at the table‐wide α' = 0.01 level for multiple comparisons.

### 2.5 Gene flow analysis

Several demes were geographically proximate but separated by potential barriers, such as the Derwent River and Tamar Valley, and it was therefore important to assess potential migration events, both recent and historical. We used BayesAss3 v3.0.4 (Wilson & Rannala, 2003) and BA3‐SNPs v1.1 (Mussmann et al., 2019) to estimate recent migration rates between demes. BayesAss3 estimates unidirectional migration rates (m) from each deme into each other deme, while BA3‐SNPs automates the parameter selection and burn‐in for BayesAss3 using Markov Chain Monte Carlo methods and allows BayesAss3 to be run for large SNP datasets by removing the upper limit on number of loci. Lower mean estimates were visualised with the R package pheatmap v1.0.12 (Kolde, 2019). Notably, BayesAss3 only estimates migration ‘over the last several generations’ between the recent inferred parents of the samples provided (Wilson & Rannala, 2003).

Maximum likelihood of ancestral migration and genetic drift of ancestral populations was estimated with TreeMix v1.13 (Pickrell & Pritchard, 2012). To root the tree using an outgroup, we used DArTseq data from 17 *P. gunnii* from a fenced site on mainland Australia (Mt Rothwell). We did not compare heterozygosity in this population to any Tasmanian demes as the genome scaffold was generated from a Victorian population, which could lead to biased comparisons. TreeMix was run with 100 replicates for each of 0–3 migration fronts, and log‐likelihood of each run reported. Demes identified as having high levels of genetic drift by TreeMix were assessed for bottlenecks using DIYABC‐RF v1.0 (Collin et al., 2021), with 10,000 training simulations and 5000 trees. Two scenarios for each high drift deme were used, divergence from the geographically closest neighbour between 1000 and 2000 generations ago (a single generation time is approximately 2 years for this species), and divergence 1000–2000 generations ago followed by a bottleneck of Ne < 100 between 10 and 1000 generations ago.

### 2.6 Autosomal heterozygosity

We used a custom pipeline for genotyping and filtering bam files for autosomal heterozygosity (Schmidt et al., 2023). This pipeline takes a set of bam files, performs initial quality filtering, genotypes all individuals together, applies hard filters to all individuals together, then exports each individual as its own file to be filtered for sequencing depth, missing data, star alleles and spanning deletions. By genotyping all individuals together, this method allows for greater accuracy in calling rare variants, while filtering each individual separately evades issues from differential levels of missing data (Schmidt et al., 2021), low sequencing depth (Nielsen et al., 2011), high sequencing depth (Li, 2014) and polyallelic sites (Sopniewski & Catullo, pers. comm.). This pipeline also retains all sites passing these filters irrespective of polymorphism and thus will not be biased by sample size (Schmidt et al., 2021). Initial quality filtering used samtools v1.16 to remove unmapped reads and non‐primary alignments. GVCF files were produced for each individual using HaplotypeCaller in GATK v4.2.6.1 (Van der Auwera & O'Connor, 2020), and these were genotyped using GenotypeGVCFs set to ‘‐‐include‐non‐variant‐sites’. Hard filtering excluded indels then followed standard GATK guidelines for non‐model taxa, setting ‘QD < 2.0’, ‘QUAL < 30.0’, ‘SOR > 3.0’, ‘FS > 60.0’ and ‘MQ < 40.0’. Filtering of individual genotypes used bcftools v.1.16 to remove missing data sites, sites with star alleles, sites with less than 15X coverage and sites with more than 95X coverage. Polyallelic sites were atomized into multiple SNPs using ‘bcftools norm ‐a ‐‐atom‐overlaps’. Pipeline code is available at https://github.com/t‐ludovic‐schmidt/autosomal_het/. We calculated the average genome‐wide heterozygosity for each individual and *F*_IS_ for each deme. We also concatenated the contigs by size and investigated patterns of heterozygosity variation across this pseudogenome. To see how individual heterozygosities varied spatially, we used ArcGIS PRO v3.1 (Redlands, 2011) to interpolate values across space. Interpolation was run using exponential kriging with 36 neighbours.

## 3 RESULTS

### 3.1 Genetic structure

We first assessed our a priori delineation of the seven demes using three methods that assess structure without population information: PCA, Structure and FineRADstructure. PCA plots differences among individuals in Cartesian space, Structure assesses each individual's proportional assignment to a set of putative ancestral populations, and FineRADstructure groups individuals by RADtag microhaplotype similarity post hoc. Together, these provide an assessment of differences between our samples unbiased by our a priori deme assignments.

**FIGURE 2:**
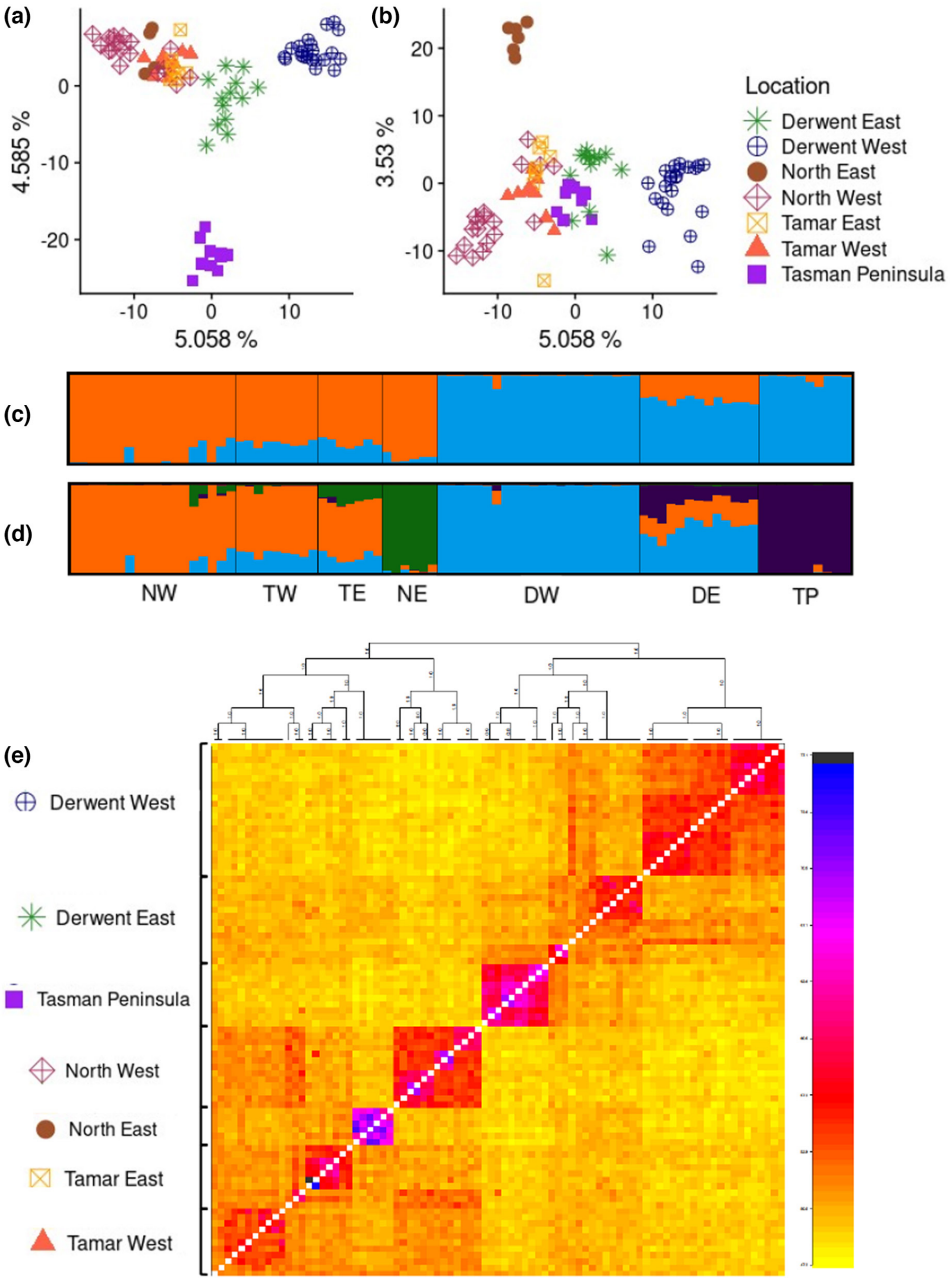
Pairwise genetic differences of 4282 SNP loci in 85 Tasmanian *Perameles gunnii*, as (a, b) Principal component analysis (PCA) with percentage of explained variation by axis 1v2 and 1v3, and (c) *K* = 2 and (d) *K* = 4 using 3020 SNPs after thinning to one polymorphism per 1000 base pairs to remove linkage. Structure Harvester indicated *K* = 2 had the largest Δ*K*, while *K* = 4 had a slightly positive Δ*K* (Table S2). (e) Heatmap of coancestry coefficients generated by FineRADstructure. Cells indicate an estimate of shared coancestry between two individuals and are shaded from yellow (low) to blue (high) to indicate increasing levels of coancestry. Above is a clustering dendrogram based on the matrix of coancestry coefficients, estimating deme divergence points, posterior probability of all arms = 1. DE, Derwent East; DW, Derwent West; NE, North East; NW, North West; TE, Tamar East; TP, Tasman Peninsula; TW, Tamar West.

The first component of the PCA (Figure 2a,b) described a continuum of variation within and between the northern samples and the southern samples, while components two and three reflected the clustering of the Tasman Peninsula and North East samples respectively (Figure 2a,b). Further components did not indicate any additional clusters. For Structure analysis (Figures 2c,d), Structure Harvester indicated a strongly positive Δ*K* at *K* = 2 and weakly positive *K* = 4 (Table S2), likely reflective of two levels of hierarchical structure (Evanno et al., 2005). While Structure is very accurate at the highest hierarchical level, it is weak at elucidating genetic structure at lower levels (Evanno et al., 2005). While sub‐setting our data into the two major clusters for reanalysis would remove this top level of hierarchy and increase accuracy (Evanno et al., 2005), Δ*K* cannot suggest fewer than two probable clusters, and so subsetting in this way would affirm at least two clusters in each subset, regardless of whether this is biologically representative. We therefore decided to present this data using all samples at both levels of *K*, to show both the higher and lower hierarchical structure.

The upper hierarchical level describes the pre‐established north–south divide in this species (Figure 2c), while the lower level exposes the isolation of the Tasman Peninsula and the North East within these areas (Figure 2d). Derwent East appears in Structure as highly admixed, possibly reflecting a historical connection between the three adjacent demes it shares clusters with. Contemporary landscape distribution of *P. gunnii* (Figure 1b) means gene flow is unlikely between the south and the north, and other analyses indicate it is unlikely to be highly admixed with the Tasman Peninsula. Three Derwent East samples had higher rates of clustering assignment with the Tasman Peninsula (Figure 2d), and were also geographically close to the Tasman Peninsula but behind a significant migration barrier, the Denison Canal.

The FineRADstructure (Figure 2e) analysis was able to sort the vast majority of samples into seven demes that were almost identical to our a priori groupings, indicating that there is discrete genetic structure among these demes. The North East and Tasman Peninsula demes had highest internal coancestry, and both demes clustered within broader clusters of the north and south, respectively, suggesting that the differentiation of the North East and Tasman Peninsula observed in the PCA is the result of some combination of genetic drift and inbreeding. The North West and Derwent West also had high internal coancestry and low coancestry with neighbouring areas. Tamar West was perhaps the least clearly partitioned in this analysis, with many individuals showing a moderate level of coancestry with individuals from the North West. Five samples assigned to the North West a priori were sorted with the Tamar West (2) and Tamar East (3) demes, yet showed high coancestry with other North West samples, likely due to the North West's geographic spread and proximity of these samples to both Tamar demes (Figure 1). Three samples sorted into Derwent East had high coancestry among themselves, and moderate coancestry with other Derwent East and Tasman Peninsula samples. These three samples were the same as highlighted in the Structure analysis (Figure 2d), located on the border of the Derwent East and Tasman Peninsula demes and <1 km north of a major migration barrier. Two sub‐groups appeared within Derwent West, suggesting minor substructure within this deme that appeared to occur across the Huon River. One individual from Derwent West was sorted into Derwent East, yet still showed high coancestry with Derwent West, and showed a similar clustering pattern as Derwent East in the Structure analysis (Figure 2d). This individual was sampled from an isolated park in downtown metropolitan Hobart (Queens Domain) and may in fact represent an animal that was moved from Derwent East to Derwent West.

**FIGURE 3:**
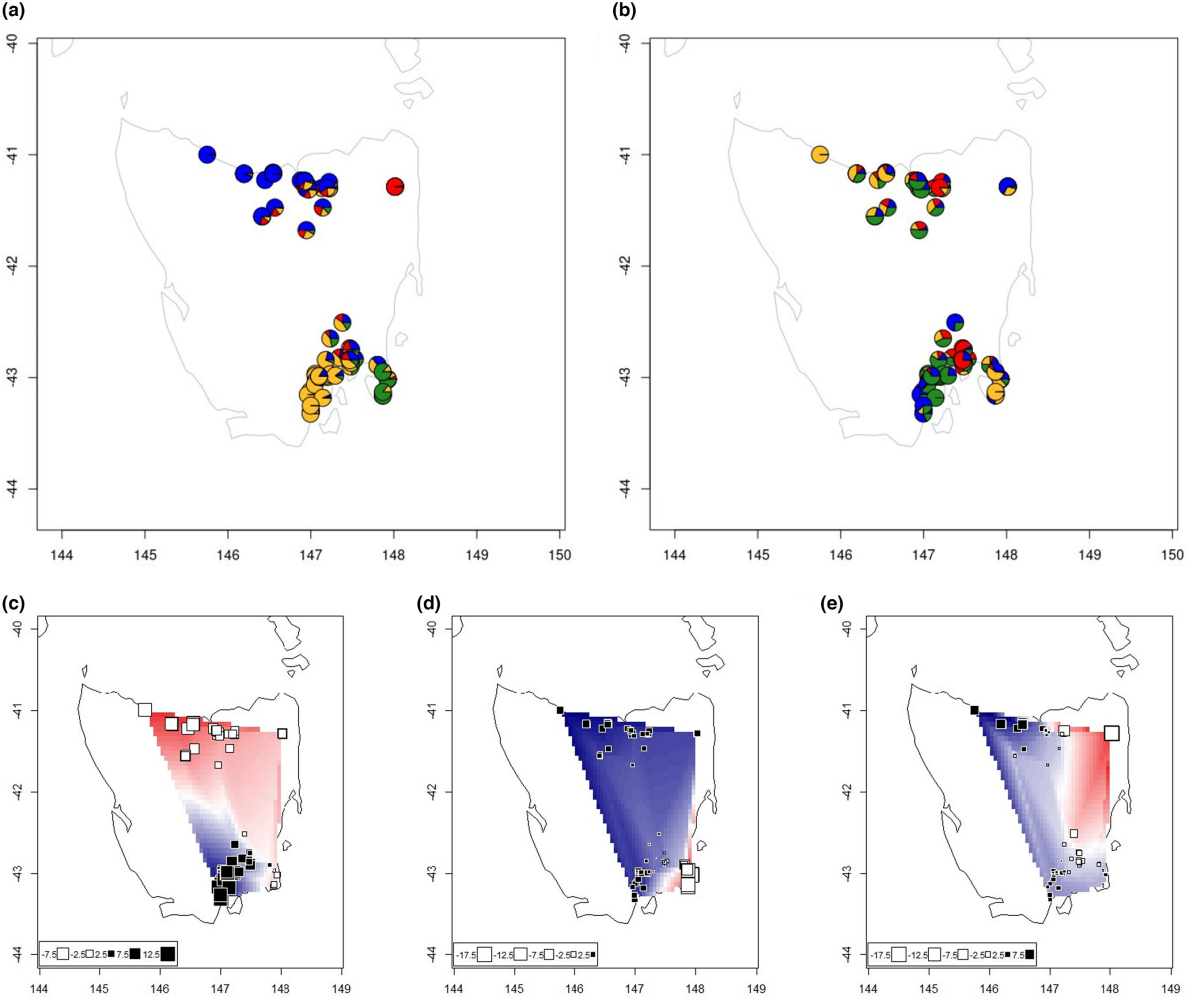
(a) ConStruct (*K* = 4) non‐spatial analysis where genotype similarity decay over distance is not accounted for, with each colour representing a distinct genetic cluster. (b) ConStruct (*K* = 4) spatial analysis where decay in individual genotype similarities across space is accounted for. Sites with genotype similarity explained by spatial decay will display similar clustering patters. (c–e) spatial structure of genotypes in sPCA for the three principal components identified as displaying global structure (Figure S4), plotted as points showing individual loading for each of three principal components, and spatially interpolated shading.

Having identified discrete patterns of genetic structure, we assessed continuous patterns using Mantel tests, ConStruct and sPCA. Mantel tests found significant isolation by distance between demes, and within all demes apart from Tamar West and North East (Figure S3). ConStruct used *K* = 4 to compare two analyses, one incorporating a spatial decay component and one that is purely discrete (non‐spatial). *K* = 4 was the value used for Structure, and it was also at the plateau of predictive power for ConStruct (Figure S4a). In the non‐spatial model (Figure 3a) clustering was similar to the results from Structure, and this discrete structure remained even when a spatial decay component was included (Figure 3b), which also indicated fine‐scale differences across the Tamar and Derwent Rivers. sPCA identified three statistically significant global genetic structures (Figure S4b, *p* = .001 at 1000 permutations). These structures indicated a north–south divide (Figure 3c), strong local differentiation between the Tasman Peninsula and Derwent East (Figure 3d), and differentiation of the North East with a steep cline across the Tamar West and Tamar East demes (Figure 3e). This steep genotype differentiation across distances of less than 5 km, coupled with results from ConStruct and FineRADstructure, suggests that global genetic patterns are structured by discrete patterns as well as isolation by distance.

The North East and the Tasman Peninsula had the highest mean *F*_ST_ to other demes, which were 30% and 26% higher, respectively, than the next highest (Derwent West) (Table 1). Pairwise genetic distances were low between Derwent East and the North West, Tamar West and Tamar East demes, despite the Derwent East's separation of at least 150 km from the northern samples, reflecting background clustering observed in Structure (Figure 2d). Outside of the North East and the Tasman Peninsula, pairwise *F*_ST_ was low among northern and southern regions (Table 1).

**TABLE 1:** Weir–Cockerham pairwise *F*_ST_ and private alleles for each group at 4282 SNP loci in 85 Tasmanian *Perameles gunnii* samples, generated with the package StAMPP (*F*_ST_) and Stacks2 (private alleles).

*F*_ST_ (*n*)	NW	TW	TE	NE	DW	DE	TP	Pop. Mean
NW (18)								0.1186
TW (9)	0.0603							0.1190
TE (7)	0.0727	0.0771						0.1224
NE (6)	0.1812	0.1903	0.1818					0.2050
DW (22)	0.1299	0.1205	0.1263	0.2192				0.1445
DE (13)	0.0880	0.0802	0.0876	0.1680	0.0792			0.1070
TP (10)	0.1795	0.1858	0.1888	0.2894	0.1922	0.1387		0.1957

*Note*: Darker shading indicates higher *F*_ST_ values, generated in Microsoft Excel. All values were statistically significant at the table‐wide α' = 0.01 level after Bonferroni correction for multiple comparisons.

Abbreviations: DE, Derwent East; DW, Derwent West; NE, North East; NW, North West; TE, Tamar East; TP, Tasman Peninsula; TW, Tamar West.

### 3.2 Gene flow

**FIGURE 4:**
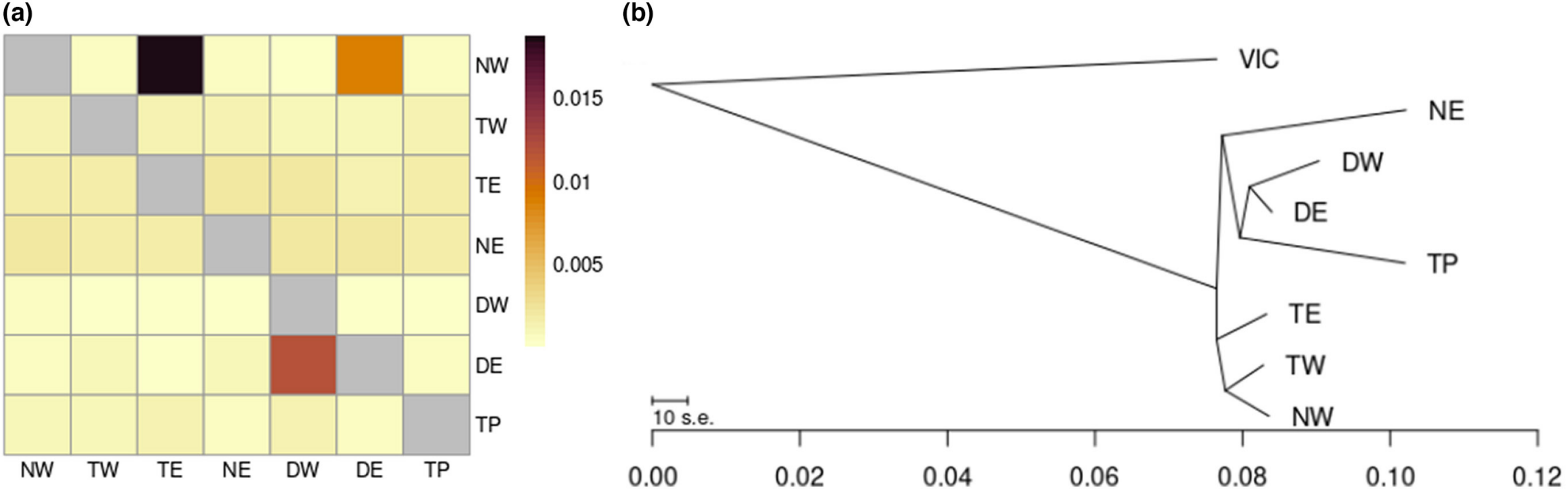
(a) Heatmap of mean posterior estimates of recent migration between seven Tasmanian *Perameles gunnii* demes using BayesAss3 with 4282 SNPs. Donor/source demes are on the *x*‐axis, and recipient/sink demes on the *y*‐axis. Numerical values of lower mean estimates available in Table S3. (b) TreeMix dendrogram of relative drift between demes and a Victorian (mainland Australia) outgroup, with 0 migration fronts. DE, Derwent East; DW, Derwent West; NE, North East; NW, North West; TE, Tamar East; TP, Tasman Peninsula; TW, Tamar West; VIC, mainland Australia outgroup.

Using BayesAss3 and BA3‐SNPs, we estimated a small amount of migration from the Tamar East into the North West at or above 2.1% of parents of the last several generations, and from Derwent West into Derwent East at or above 1.2% (Figure 4a, Table S3). These pairs both share geographically proximal sites (Figure 1), and it is likely that there may be migration between them in areas not covered by our sampling regime. We also observed a marginal amount of migration from Derwent East to North West. As these demes contained two locations that encroach on the now largely uninhabited Midlands region, this may be an indication of migration from when connectivity still existed in the late 1980s or early 1990s (Mallick et al., 1998). However, the overall findings of this analysis indicate minimal movement of individuals, consistent with current knowledge about the dispersal characteristics of *P. gunnii*.

TreeMix migration fronts were generally placed between the Victorian outgroup and the North West or Tasman Peninsula demes (Figure S2), suggesting that it is unlikely any large, historical migration has occurred between any of the demes. The maximum likelihood tree reflects patterns found in our other analyses, with an initial north–south divide followed by further partitioning of demes within these areas (Figure 4b). The North East appears to have become isolated from the other northern demes at a similar time as the southern demes, reinforcing its apparent isolation, and the North East and Tasman Peninsula appear to have undergone the highest degree of genetic drift, as shown by the length of the tree branches (Figure 4b). DIYABC found no evidence that the Tasman Peninsula and North East had undergone recent population bottlenecks of N_e_ < 100 (Table S4), suggesting that these patterns are better explained by population separation than a recent founder effect.

### 3.3 Autosomal heterozygosity

**FIGURE 5:**
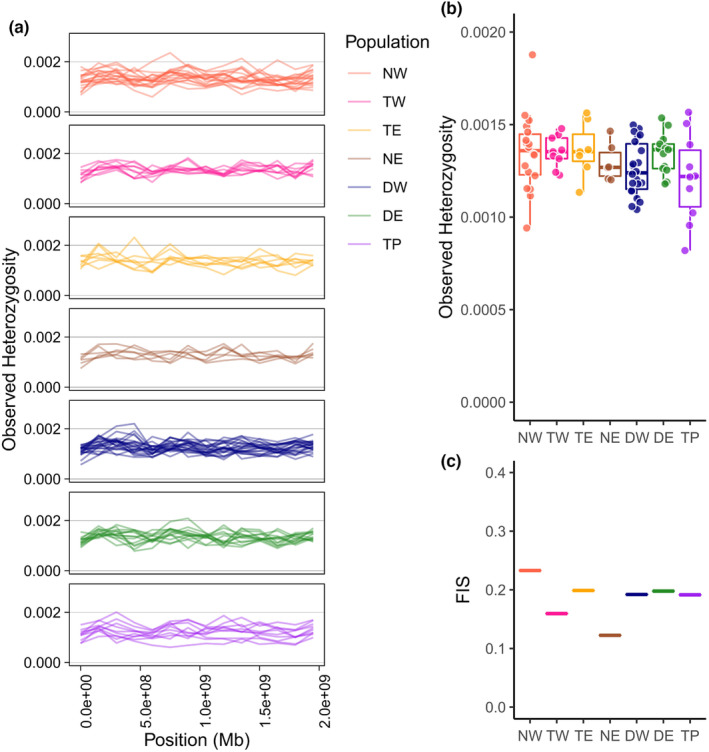
Genome‐wide (autosomal) observed heterozygosity and Wright's inbreeding coefficient (*F*_IS_) of 85 Tasmanian eastern barred bandicoot. (a) Autosomal heterozygosity for individual samples across all scaffold positions, (b) mean autosomal heterozygosity for demes and (c) mean Wright's Inbreeding Coefficient (*F*_IS_) per deme, calculated by GATK. DE, Derwent East; DW, Derwent West; NE, North East; NW, North West; TE, Tamar East; TP, Tasman Peninsula; TW, Tamar West.

Observed autosomal heterozygosity (H_O_) was calculated for each individual separately, using all sites with confidently called genotypes after filtering each individual for coverage and missing data. This reduced concerns around differential data quality between roadkill tissue samples and higher quality live‐trapped tissue samples. This left between 811,930 and 2,118,697 sites for each individual. There were no correlations between H_O_ and the number of retained sites (*R* ^2^ = .037) or the sampling year (*R* ^2^ = .025). H_O_ was roughly consistent across the concatenated pseudogenome (Figure 5a) but varied considerably among individuals, from 8.19 × 10^−4^ in an individual from the Tasman Peninsula (TP) to 1.88 × 10^−3^ in an individual from the North West (NW) (Figure 5b). Mean H_O_ was slightly lower in three demes (Figure 5b, Table S5). However, variance within demes was large, particularly in the Tasman Peninsula and North West where the ratios between the highest and lowest H_O_ were 1.99 and 1.91 respectively. Due to this, a single factor ANOVA indicated no significant difference in autosomal H_O_ among demes (*F*_6,78_ = 1.438, *p* = .211; Table S6).

**FIGURE 6:**
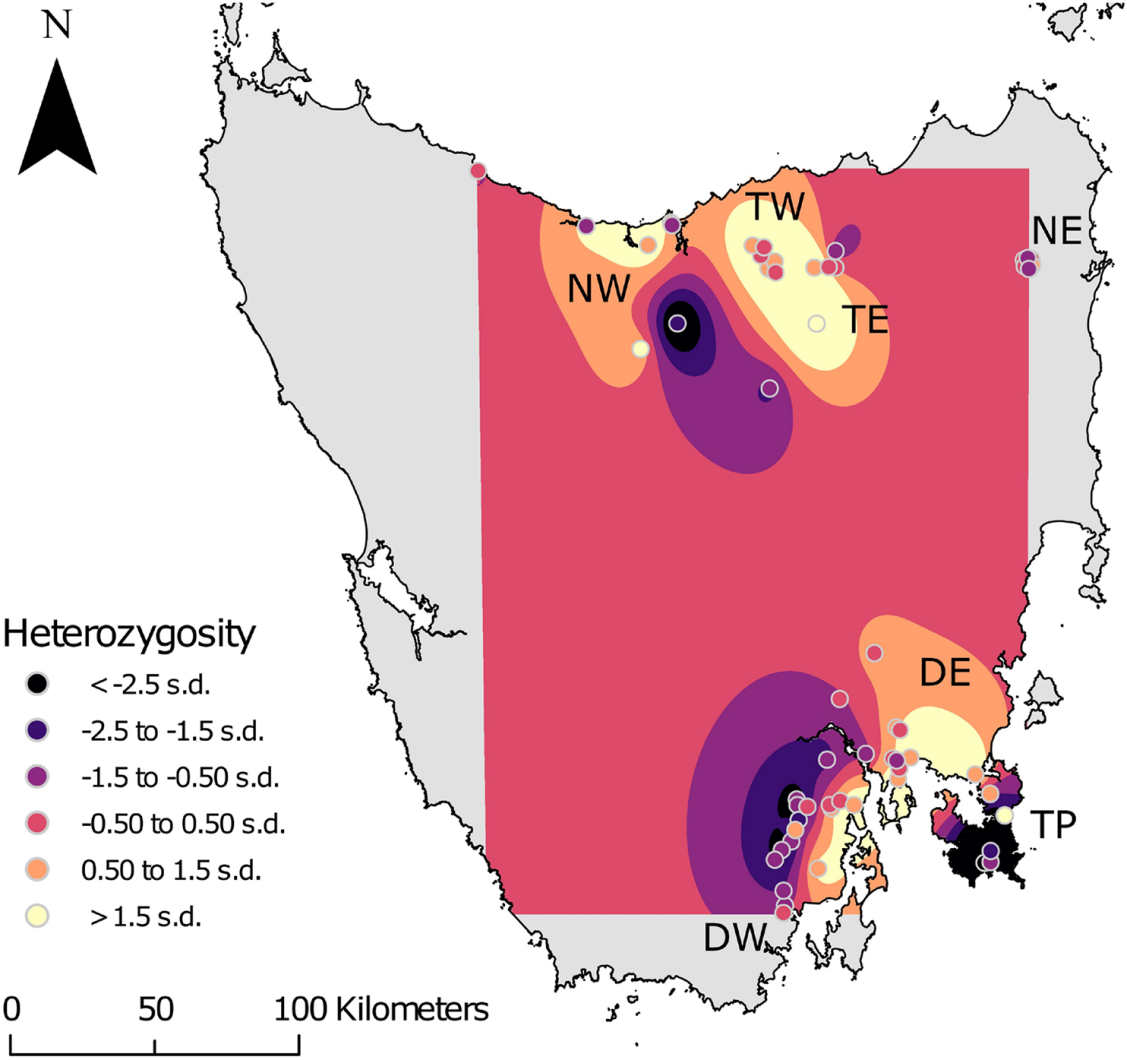
Exponential kriging interpolation of 85 individual *Perameles gunnii* autosomal heterozygosity on a Goode projection map of Tasmania, with indicative deme markers. Shading indicates an area's predicted deviation from global mean heterozygosity, measured in standard deviations (s.d.). DE, Derwent East; DW, Derwent West; NE, North East; NW, North West; TE, Tamar East; TP, Tasman Peninsula; TW, Tamar West.

Spatial interpolation of H_O_ via exponential kriging helped clarify these patterns (Figure 6). Here, the Tasman Peninsula was identified as a region where individuals with much lower H_O_ than average were more common. While the Tasman Peninsula had one individual with the second highest H_O_, it also had three of the four lowest scores (Figure 5b). Two of these individuals were in the southern section of this region near Port Arthur, while a third was located in Murdunna in the northern section. Another sample of low heterozygosity was located in the inner northern region of the North West, adjacent to an exceptionally high‐heterozygosity sample (Figures 5a–c and 6). There was no obvious pattern of higher heterozygosity cores and lower heterozygosity edges that can occur with spatial distributions at low neighbourhood sizes (Battey et al., 2020).

Wright's inbreeding coefficient (*F*_IS_) was broadly reflective of the geographic distance that samples were accumulated across. The lowest *F*_IS_ was in the North East, where samples were all located near a single township with approximate location data only, and in Tamar West (TW), where all samples were within 10 km of all other samples (Figure 5c). Higher *F*_IS_ is likely indicative of non‐random mating due to the distances between sampled areas, and should not be interpreted as a metric of inbreeding directly.

## 4 DISCUSSION

The preeminent pattern of population structure in Tasmanian *P. gunnii* is a north–south divide following the apparent extinction of populations in midland areas around 1989, which has previously been established through roadkill observations (Driessen et al., 1996; Mallick et al., 1998) and genetically with microsatellites, VNTR and mtDNA (Robinson, 1995; Robinson et al., 1993; Weeks et al., 2013). Our findings are consistent with these observations, and we further identify patterns of discrete and continuous population genetic structure among demes in the north and the south. While our seven a priori demes showed patterns of isolation by distance, this alone is insufficient to explain the genetic structure we observed, and we found discrete clusters of individuals along the lines of our a priori assignments that have had little recent gene flow among them. This indicates that *P. gunnii* is sensitive to geographical isolation, congruent with the low dispersal ability of this species. Critically, we identify two isolated demes experiencing high genetic drift in the North East and the Tasman Peninsula. We found substantial variation in heterozygosity among individuals within demes, and in the Tasman Peninsula there was an almost twofold difference between the most to the least heterozygous individuals. Despite this heterogeneity, spatial interpolation of individual autosomal heterozygosities indicated that there was an abundance of low‐heterozygosity individuals in the southern edge of the Tasman Peninsula and western edge of Derwent West.

Few studies have explicitly considered the relationship between genetic structure and variation in individual heterozygosity. Our findings here suggest that even small populations that are highly differentiated and isolated from others may not have a level of heterozygosity specific to individuals of that population. It follows that, when heterozygosity estimates are based on single individuals sampled from variable populations such as the North West or the Tasman Peninsula, there could be dramatically different downstream inferences depending on which individuals are sampled. These findings thus support genomic approaches that sample and sequence many individuals from across a population's range. Additionally, while autosomal heterozygosity is generally highly correlated with runs of homozygosity (Kardos et al., 2018, 2021; Mathur et al., 2023), it can also be calculated from highly fragmented reference assemblies. The largest contig in our *P. gunnii* assembly was 407,879 bp, which limits inferences from runs of homozygosity larger than this but is suitable for estimating autosomal heterozygosity.

Although the Tasman Peninsula contained several individuals of above average heterozygosity, it also contained three of the four lowest heterozygosity individuals. Establishment of a dispersal barrier in 1905 (the Denison Canal) suggests the Tasman Peninsula is experiencing recent yet strong isolation, and patterns of variable heterozygosity may hold clues into how heterozygosity loss emerges in a population; in this case, that average and high heterozygosity individuals remain in the population even while individuals begin to accumulate at the lower bound of heterozygosity. This is also reflected in Derwent West, where higher heterozygosity individuals clustered on the eastern side of the Huon River, and lower heterozygosity individuals clustered on the western side. Similar patterns of genetic structure were identified in Derwent West by FineRADstructure only, posing the question of whether structured variation in individual heterozygosity arises before, after, or with discrete genetic structure. Further studies into the relationship between structure and heterozygosity are required to see if this pattern is repeated in other systems, where it could serve as an early warning sign of a population in trouble from drift and inbreeding. The spatial and geographic barrier elements are likely to be important here and in other studies, as dispersal rates will determine how well‐mixed a given population is, which should also influence the degree of variation in individual autosomal heterozygosities.

The pattern of north–south genetic isolation found in *P. gunnii* is relatively unique among Tasmanian marsupials with published genetic analysis. Tasmanian devils (*Sarcophilus harrisii*, Boitard 1841) were previously found to have widespread gene‐flow, though with an outlying north‐western population (Jones et al., 2004), and more recently have been structured by the advance of two devil facial tumour diseases, with greater contemporary structure appearing in the north‐east (Farquharson et al., 2022; Miller et al., 2011). Eastern quolls meanwhile are largely structured by geographic distance, with mixing between nearby regional clusters (Cardoso et al., 2014). Eastern bettong (*Bettongia gaimardi*, Desmarest 1882) distribution is heavily influenced by precipitation resulting in low genetic differentiation in core areas and higher differentiation at edge populations, and long‐nosed potoroos (*Potorous tridactylus*, Kerr 1792) are broadly similar among north and south populations though some are isolated (Frankham et al., 2016; Proft et al., 2021). While *P. tridactylus* shares a similar diet and habitat preference with *P. gunnii, P. tridactylus* is approximately twice as large and presumably more mobile, potentially reducing any effects of habitat fragmentation on its population structure. A panel of experts found *P. gunnii* marginally more susceptible to feral cat predation than *P. tridactylus*, although both were ranked as having at least ‘High’ vulnerability (Radford et al., 2018).

*Perameles gunnii* inhabiting the Tasman Peninsula appear to be fragmented from mainland Tasmania by a barrier established only 120 years ago: the Denison Canal, a 7 m wide, 900 m long waterway crossable by a single narrow vehicle bridge. The Tasman Peninsula and Derwent East contained samples within 1 km of each other across this barrier, yet a strong population structure was observed between these samples. The Tasman Peninsula had the lowest pairwise *F*_ST_ with Derwent East, and three proximal Derwent East samples shared high levels of Tasman Peninsula ancestry, but no recent gene flow was evident. The Denison Canal therefore appears to be a relatively impermeable dispersal barrier. By comparison, modest differentiation occurred across the Derwent River, between Derwent West and Derwent East. The efficacy of these barriers appears not as strong as the Denison Canal, suggesting that gene flow may be occurring at upstream sites outside of urban areas, but making proximal samples differentiated (Łopucki & Kitowski, 2017). In the Tamar demes, a small amount of differentiation was observed that was not explicable by distance alone, suggesting that the Tamar River and Launceston also act as modest barriers. The FineRADstructure and ConStruct analyses also both detected slight differentiation among individuals across the Huon River, though it was unclear whether these should be treated as distinct genetic groups. This minor genetic differentiation across the Huon River is more clearly observed by the structuring of individual heterozygosities, which is higher on the eastern side of the river than the west, suggesting less genetic mixing across the region.

The North East, represented by six samples from Pyengana, was another highly differentiated deme. Unfortunately, geolocation data were not available to estimate geographic distances between these samples, and we can only use the six samples as a proxy for the broader area. This deme shows a high degree of separation from the proximal Tamar demes, even when allowing for isolation by distance, and samples had extremely high coancestry among themselves. The North East also had relatively low population mean heterozygosity and low variation in individual heterozygosities. Low heterogeneity in individual heterozygosity and low *F*_IS_ supports our assumption that all samples came from a very small area, or that all sampled animals inhabited a similar section of a larger populated area. However, whether this represents a population edge, a node in a network of demes, or an isolated fragment remains unclear from these data alone. The prevailing habitat across north‐eastern Tasmania is that of mountainous, wet woodlands, unsuitable for *P. gunnii*, interspersed with agricultural properties which represent marginally more suitable habitat, suggesting that a network of small demes is plausible; however, this does not rule out complete isolation, which is plausible given the high levels of drift observed in TreeMix. If Pyengana is part of a network of demes in this region then it may be secure, provided migration pathways between refugia can be preserved (Hoffmann et al., 2015), however extensive further sampling in this region is required.

Derwent East showed genetic similarity with both northern and southern demes, but no evidence of recent migration. *Perameles gunnii* population connectivity between Launceston and Hobart still existed until shortly before 1989 (Robinson et al., 1991), and the similarity between Derwent East and the northern demes suggests a recently severed isolation by distance pattern, with differences yet to be exacerbated by genetic drift. Many demes had samples separated by >50 km, well above the assumed generational dispersal ability for a bandicoot (Piggott et al., 2018; Robinson et al., 2018), and the high *F*_IS_ seen in geographically larger demes suggests a continuous‐space Wahlund effect linked to spatial structure within demes (Battey et al., 2020). North East and Derwent West were the only demes where samples were within a single generation's assumed maximum dispersal, and consequently the sites with the lowest *F*_IS_.

Future work involving extensive sampling in each deme could help clarify the fine scale patterns of population structure we observed. This could also reveal how structure and genetic diversity has changed since 2011, when the samples for this study were collected. This is particularly important for the two isolated demes we identified, which may have experienced increased inbreeding in the six or more generations since 2011. An evaluation of population trends has seen continuing declines in Tasmania during this time (National Environmental Science Program – Threatened Species Research Hub, 2019). Additional sampling of all Tasmanian populations would be useful here, with priority assigned to the two isolated demes we identified, as well as the sampling gap between the Tamar and North East regions.

Our pipeline for calculating individual autosomal heterozygosities may be useful when associating individual fitness with heterozygosity or for selecting animals at the individual or population level when effecting species translocations. Despite widespread acceptance of the effects of isolation, small population size and low diversity on population fitness, fears of outbreeding depression have stymied the implementation of genetic mixing strategies (Liddell et al., 2021; Meek et al., 2015). This causes uncertainty for conservation managers, who are often hesitant to implement mixing strategies due to the perceived possibility of causing harm (Meek et al., 2015; Weeks et al., 2011) or from a lack of understanding around scientific concepts, data interpretation or recommended best‐practice (Cook & Sgrò, 2017, 2019; Meek et al., 2015; Ralls et al., 2018). This is despite mounting evidence of the beneficial outcomes of genetic restoration and growing calls for this to be a go‐to course of action (Bell et al., 2019; Frankham, 2015; Hoffmann et al., 2015, 2021; Ralls et al., 2020; Stewart et al., 2017; Weeks et al., 2011; Whiteley et al., 2015). Here, we show that using individual autosomal heterozygosity to examine heterogeneity in population heterozygosity could provide valuable information for selecting potential source populations when undertaking a genetic restoration, as it allows us to select source populations with uniformly high heterozygosity, or even individuals with exceptional heterozygosity. However, this data should go hand‐in‐hand with other useful metrics such as the number of private alleles and *F*_ST_, which can be used to estimate the number of novel alleles a source population can provide.

## 5 CONCLUSION

Wild populations can display spatial patterns of genetic structure that are continuous as well as discrete. Understanding these patterns is vital for planning conservation and management strategies and for accurately estimating other genetic parameters such as individual heterozygosity. Here, in the first genomic study of Tasmanian *P. gunnii*, we observed discrete genetic structure across geographical barriers as well as continuous isolation by distance patterns, and identified two genetically isolated demes. Following individual‐level analysis of genetic structure, we used a novel pipeline to estimate individual‐level autosomal heterozygosity, and showed that heterozygosity varied considerably among individuals within demes, but was lowest in the isolated Tasman Peninsula. Spatial interpolation showed that individual heterozygosity was unequally distributed in some demes, which may reflect recent or subtle barriers to gene flow not detected by other analyses. These findings point to individual‐level autosomal heterozygosity as a useful parameter for conservation genetics, particularly when samples are georeferenced and when genetic structure has complex spatial patterns. Future work could investigate whether heterozygosity loss in specific individuals can be used to detect recent population contractions or fragmentation. Future research should also aim to understand the processes that lead to large differences in heterozygosity among neighbouring individuals such as we have observed in Tasmanian *P. gunnii*. Finally, urgent and extensive genetic sampling should occur around the two isolated demes identified in the Tasman Peninsula and Pyengana (North East). Genomic data from these regions dates to 2008, and active management intervention may be required if demographic declines have continued in either location.

## AUTHOR CONTRIBUTIONS

Ary A. Hoffmann and Andrew R. Weeks conceptualised this study. Andrew R. Weeks designed this study. Dean Heinze and Robbie Gaffney collected or oversaw the collection all Tasmanian samples. John G. Black and Anthony R. J. van Rooyen processed samples. John G. Black and Thomas L. Schmidt analysed and interpreted results. John G. Black and Thomas L. Schmidt wrote the manuscript. Thomas L. Schmidt, Anthony R. J. van Rooyen, Ary A. Hoffmann and Andrew R. Weeks provided supervision. All authors edited the final manuscript and gave final approval for publication.

## FUNDING INFORMATION

Funding for genetic analysis of samples was provided by Cesar Australia. John Black was funded by a PhD scholarship from the University of Melbourne. Thomas L Schmidt was funded by an ARC DECRA Fellowship (DE230100257). Andrew Weeks and Ary Hoffmann received financial support from the National Environment Science Program Threatened Species Recovery Hub (Federal Department of Environment and Energy) and the Australian Research Council Discovery grant scheme (DP160100661) for sequencing and assembling the genome scaffold for *P. gunnii* with ethics approval 1613835.1.

## CONFLICT OF INTEREST STATEMENT

The authors have no conflicts of interest to declare that are relevant to the content of this article.

## BENEFIT‐SHARING STATEMENT

This project established a collaboration between institutes on mainland Australia and the Tasmanian Department of Natural Resources and Environment through author Robbie Gaffney, and insights from this work will be implemented in management plans for *Perameles gunnii* overseen by this department. Benefits from this research will also accrue from the sharing of our data and results on public databases, and from the public availability and demonstration of our autosomal heterozygosity pipeline, which allows researchers to reprocess and reanalyse ours and other public datasets for comparative conservation assessments.

## Supporting information


Appendix S1.

## Data Availability

Genetic data and sample metadata will be made available on DataDryad DOI: 10.5061/dryad.ksn02v7b0.
