# Supplementary material for: Heterogeneous patterns of heterozygosity loss in isolated populations of the threatened eastern barred bandicoot (Perameles gunnii)
Source: Mol Ecol. 2023 Nov 28;33(20):e17224. doi: 10.1111/mec.17224 (PMC13084969; doi:10.1111/mec.17224)
Supplement: Supplementary file 1 — Appendix S1. [file MEC-33-e17224-s001.docx]

# Supplementary Data Tables and Figures

Supplementary data table 1: FASTA summary statistics for a draft *Perameles gunnii* scaffold based on a Victorian specimen.

| Statistic |  |
| --- | --- |
| Species | *Perameles gunnii* |
| Population | Victorian |
| Sequencing method | PacBio and Illumina |
| Assembly level | Scaffold |
| Scaffold L50 | 9636 |
| Scaffold N50 | 80265 |
| Scaffold L90 | 23119 |
| Scaffold N90 | 54133 |
| Scaffold len_max | 407879 |
| Scaffold len_min | 50001 |
| Scaffold len_mean | 80022 |
| Scaffold len_median | 69291 |
| Scaffold len_std | 32780 |
| Scaffold num_A | 685913098 |
| Scaffold num_T | 686488279 |
| Scaffold num_C | 390345222 |
| Scaffold num_G | 390348393 |
| Scaffold num_N | 33532460 |
| Scaffold num_bp | 2186627452 |
| Scaffold num_bp_not_N | 2153094992 |
| Scaffold num_seq | 27325 |
| Scaffold GC content overall | 35.7 |
| Contig L50 | 22506 |
| Contig N50 | 31103 |
| Contig L90 | 68358 |
| Contig N90 | 10256 |
| Contig len_max | 183656 |
| Contig len_min | 48 |
| Contig len_mean | 18917 |
| Contig len_median | 13743 |
| Contig len_std | 17555 |
| Contig num_bp | 2153094992 |
| Contig num_seq | 113814 |
| Number of gaps | 86489 |


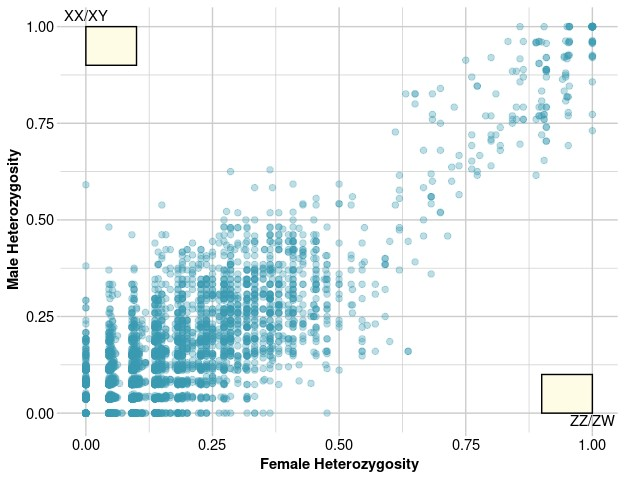

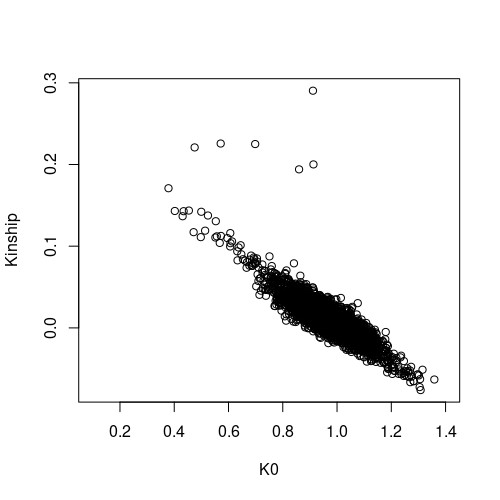

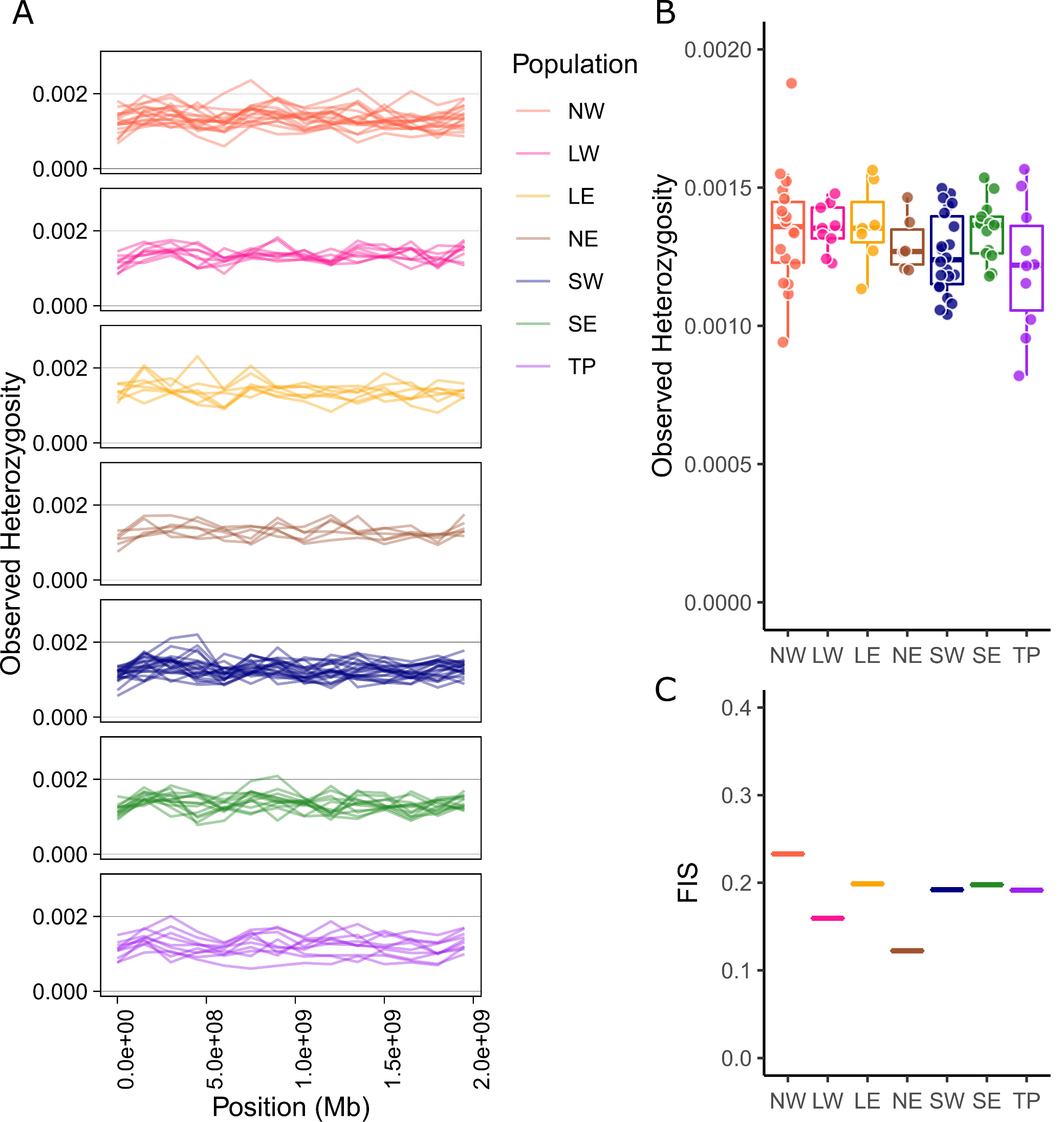

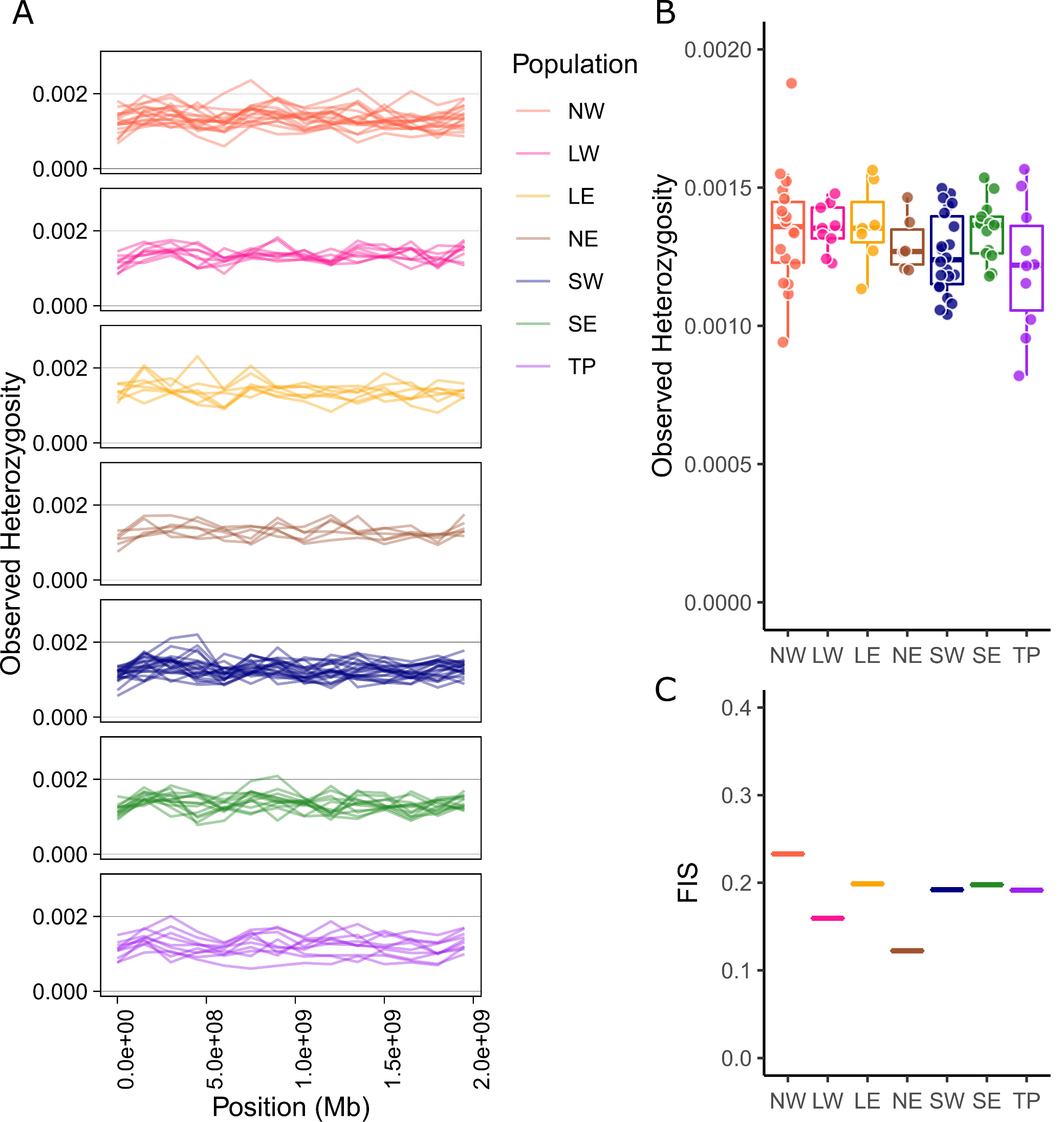


Supplementary data figure 1: Scatterplots of **A)** Estimated Kinship and probability of sharing zero alleles identical by descent (K_0_) for each sample pair of 85 *Perameles gunnii,* produced by pcrelate in the package GENESIS, and **B)** mean female and male heterozygosity for each locus in 49 *Perameles gunnii* with sex metadata, produced by gl.report.sexlinked from the package dartR. Yellow squares indicate heterozygosity values typical of sex-linked loci.

Supplementary data table 2: Structure Harvester analysis of K 1-10 in 85 Tasmanian *Perameles gunnii*. **Bold** indicates positive ΔK peaks, suggestive of biologically informative clusters.

| K | Reps | Mean LnP(K) | Stdev LnP(K) | Ln'(K) | \|Ln''(K)\| | ΔK |
| --- | --- | --- | --- | --- | --- | --- |
| 1 | 10 | -181198.1900 | 2.7209 | NA | NA | NA |
| 2 | 10 | -174558.7700 | 6.5052 | 6639.420000 | 2432.910000 | **373.993493** |
| 3 | 10 | -170352.2600 | 996.3289 | 4206.510000 | 2488.800000 | 2.497970 |
| 4 | 10 | -168634.5500 | 135.3679 | 1717.710000 | 1549.030000 | **11.443113** |
| 5 | 10 | -168465.8700 | 162.5320 | 168.680000 | 126.910000 | 0.780831 |
| 6 | 10 | -168424.1000 | 86.1312 | 41.770000 | 17.810000 | 0.206778 |
| 7 | 10 | -168364.5200 | 132.0046 | 59.580000 | 216.220000 | 1.637973 |
| 8 | 10 | -168521.1600 | 542.0123 | -156.640000 | NA | NA |

Supplementary data table 3: Mean estimates of recent migration between seven Tasmanian *Perameles gunnii* groups using BayesAss3 using 4082 SNPs. Source demes are on the top, and recipient demes on the left. **Bold** indicates migration rates of >1% at the mean posterior estimate. *Italics* indicates self-recipient migrations (along the diagonal). Abbreviations: NW = North West, TW = Tamar West, TE = Tamar East, NE = North East, DW = Derwent West, DE = Derwent East, TP = Tasman Peninsula.

|  |  |  |  |  | Source |  |  |  |
| --- | --- | --- | --- | --- | --- | --- | --- | --- |
|  |  | NW | TW | TE | NE | DW | DE | TP |
| Recipient | NW | *0.8801 (0.0313)* | 0.0132 (0.0127) | **0.0398 (0.021)** | 0.0135 (0.0129) | 0.0134 (0.0132) | 0.0268 (0.0180) | 0.0131 (0.0125) |
|  | TW | 0.0205 (0.0192) | *0.8748 (0.039)* | 0.0213 (0.0199) | 0.0209 (0.0196) | 0.0205 (0.0196) | 0.021 (0.0198) | 0.0211 (0.0198) |
|  | TE | 0.0246 (0.0227) | 0.0242 (0.0223) | *0.8567 (0.0422)* | 0.0240 (0.0219) | 0.023 (0.0209) | 0.0238 (0.0223) | 0.0237 (0.0219) |
|  | NE | 0.0264 (0.0243) | 0.0251 (0.0232) | 0.0252 (0.0233) | *0.8453 (0.0449)* | 0.0257 (0.0236) | 0.0263 (0.0243) | 0.026 (0.0243) |
|  | DW | 0.0111 (0.0106) | 0.0116 (0.0112) | 0.0121 (0.012) | 0.0119 (0.0115) | *0.9303 (0.0252)* | 0.0118 (0.0114) | 0.0112 (0.0108) |
|  | DE | 0.0167 (0.016) | 0.0164 (0.0152) | 0.0168 (0.0164) | 0.0168 (0.0158) | **0.0333 (0.0214)** | *0.8834 (0.0344)* | 0.0167 (0.0161) |
|  | TP | 0.0199 (0.0189) | 0.0193 (0.0184) | 0.0192 (0.0178) | 0.0193 (0.0185) | 0.0198 (0.0185) | 0.0202 (0.0196) | *0.8824 (0.0381)* |


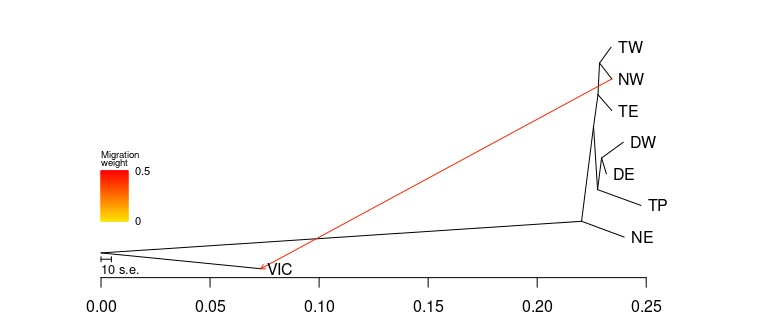

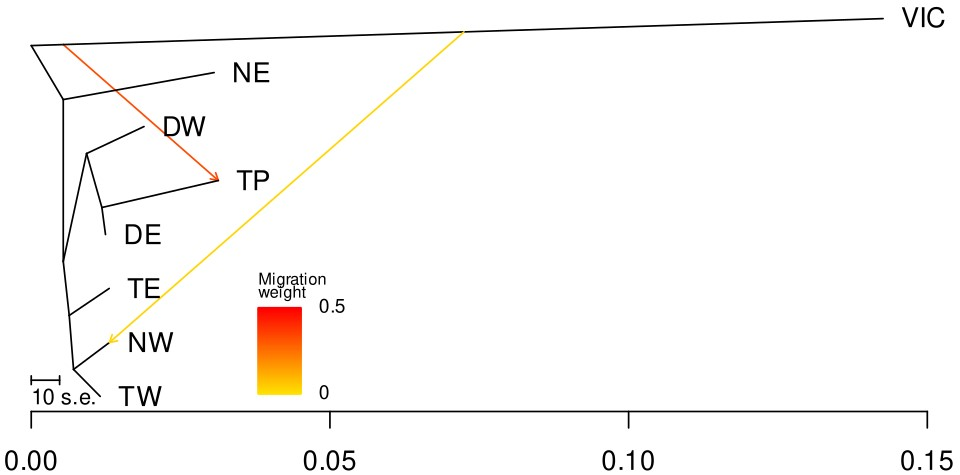

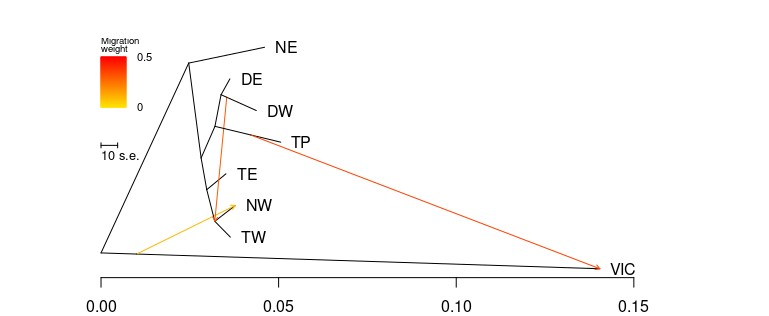

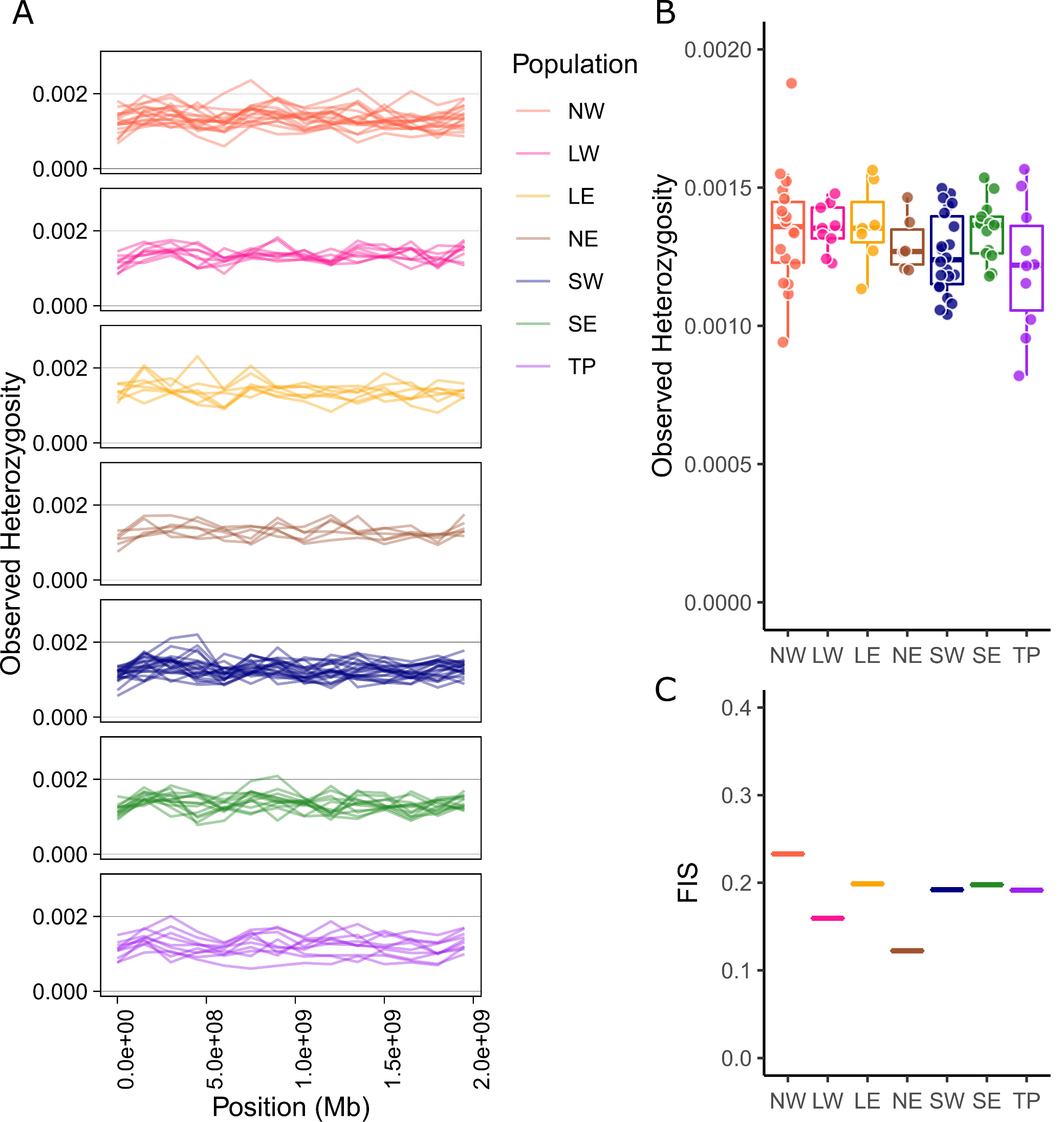

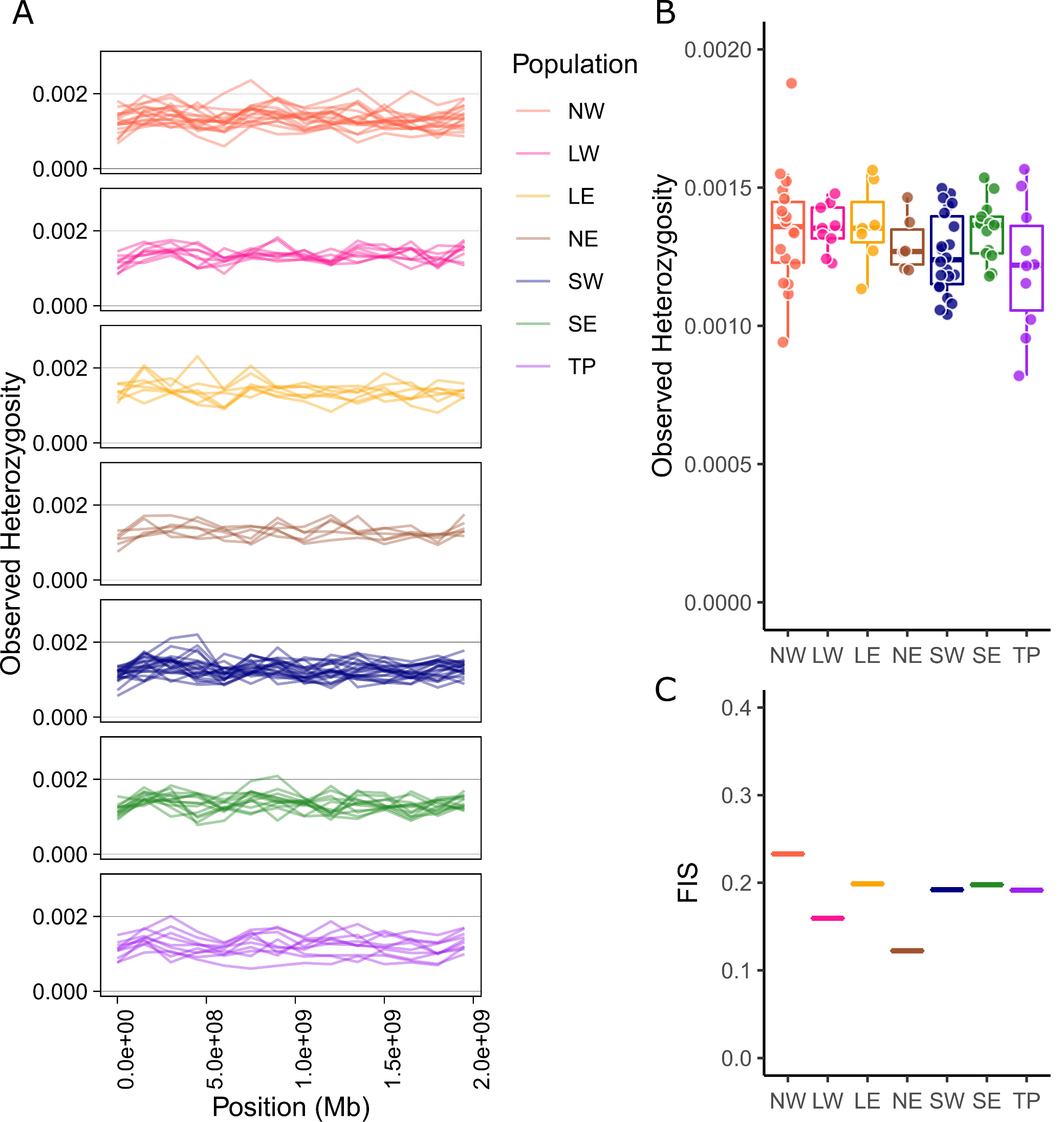

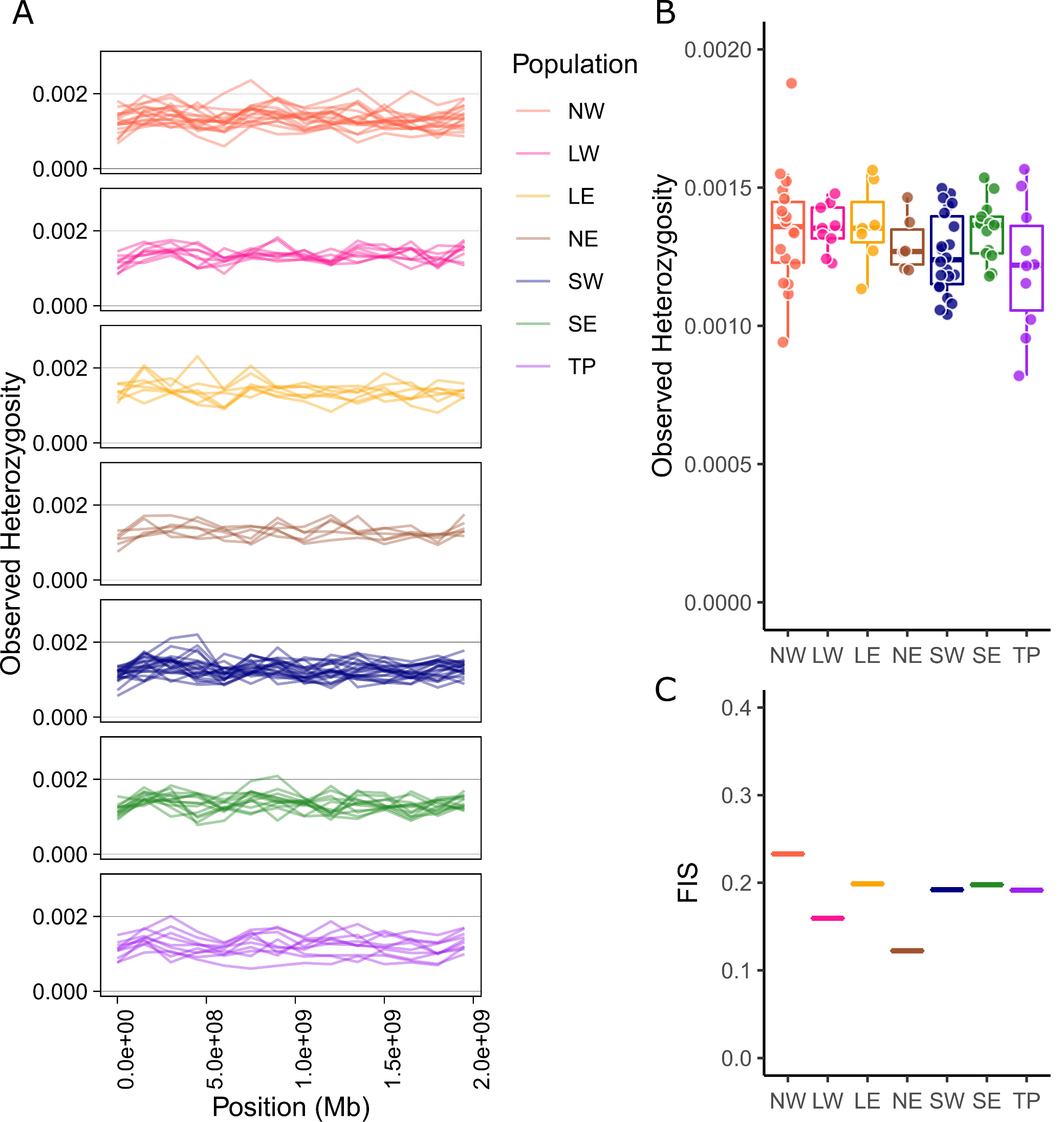


Supplementary data figure 2: TreeMix dendrogram of relative drift between Tasmanian *Perameles gunnii* groups with a Victorian outgroup, with **A)** 1, **B)** 2, and **C)** 3 migration fronts. For each number of migration fronts, the highest log-likelihood output from 100 runs is shown. Abbreviations: NW = North West, TW = Tamar West, TE = Tamar East, NE = North East, DW = Derwent West, DE = Derwent East, TP = Tasman Peninsula.

Supplementary data table 4: DIYABC output of posterior probabilities of divergence and bottleneck scenarios, run for 5,000 trees. Tasman Peninsula was assessed against Derwent East and North East was assessed against Tamar East.

|  | Divergence | Divergence + Bottleneck | Posterior Probability |
| --- | --- | --- | --- |
| North East | 4740 | 260 | 0.954 |
| Tasman Peninsula | 3881 | 1119 | 0.975 |

Supplementary data table 5: Mean observed heterozygosity (H_O_), standard deviation (SD), and standard error (SE) across 7 demes of Tasmanian *Perameles gunnii*, calculated at all autosomal sites with a depth of >15x. Cells shaded darker to indicate higher values, generated in Microsoft Excel. Estimates generated with GATK. Abbreviations: NW = North West, TW = Tamar West, TE = Tamar East, NE = North East, DW = Derwent West, DE = Derwent East, TP = Tasman Peninsula.

| Pop. | Mean H_O_ | SD | SE |
| --- | --- | --- | --- |
| NW | 0.001348 | 2.06E-04 | 4.86E-05 |
| TW | 0.001354 | 8.65E-05 | 2.88E-05 |
| TW | 0.001364 | 1.47E-04 | 5.57E-05 |
| NE | 0.001298 | 1.03E-04 | 4.20E-05 |
| DW | 0.001261 | 1.45E-04 | 3.09E-05 |
| DE | 0.001343 | 1.08E-04 | 3.00E-05 |
| TP | 0.001213 | 2.37E-04 | 7.51E-05 |

Supplementary data table 6: Single factor ANOVA of individual autosomal heterozygosities. Abbreviations: NW = North West, TW = Tamar West, TE = Tamar East, NE = North East, DW = Derwent West, DE = Derwent East, TP = Tasman Peninsula.

| SUMMARY |  |  |  |  |  |  |
| --- | --- | --- | --- | --- | --- | --- |
| *Groups* | *Count* | *Sum* | *Average* | *Variance* |  |  |
| TE | 7 | 0.00955 | 0.001364 | 2.17E-08 |  |  |
| TW | 9 | 0.012183 | 0.001354 | 7.48E-09 |  |  |
| NE | 6 | 0.007786 | 0.001298 | 1.06E-08 |  |  |
| NW | 18 | 0.024272 | 0.001348 | 4.25E-08 |  |  |
| DE | 13 | 0.017456 | 0.001343 | 1.17E-08 |  |  |
| DW | 22 | 0.027732 | 0.001261 | 2.1E-08 |  |  |
| TP | 10 | 0.012126 | 0.001213 | 5.63E-08 |  |  |
|  |  |  |  |  |  |  |
| *Source of Variation* | *SS* | *df* | *MS* | *F* | *P-value* | *F crit* |
| Between Groups | 2.27E-07 | 6 | 3.79E-08 | 1.437532 | 0.211101 | 2.217235 |
| Within Groups | 2.05E-06 | 78 | 2.63E-08 |  |  |  |
|  |  |  |  |  |  |  |
| Total | 2.28E-06 | 84 |  |  |  |  |


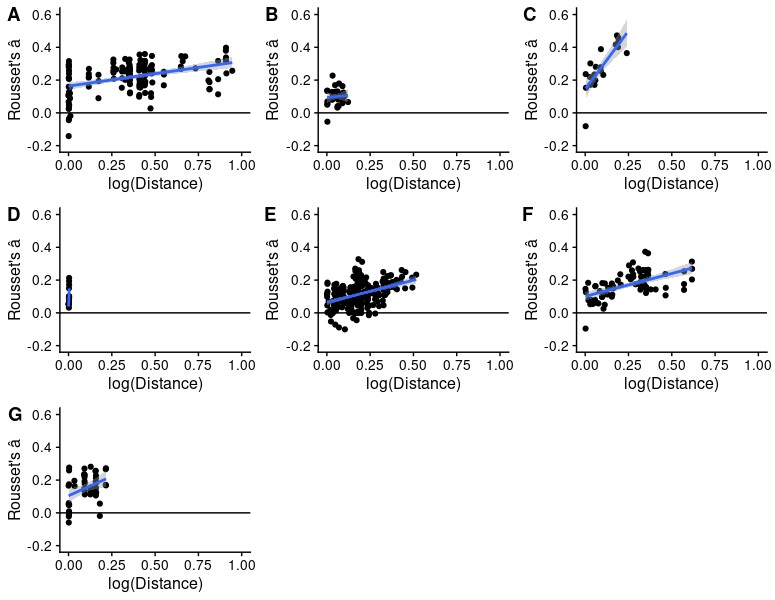


Supplementary figure 3: Rousset’s pairwise individual genetic distance, â, (Rousset, 2000) plotted against log_10_ of pairwise sample geographic distances within each *a priori* deme, fitted with a linear trendline. **A)** North West, **B)** Tamar West, **C)** Tamar East, **D)** North East, **E)** Derwent West, **F)** Derwent East, and **G)** Tasman Peninsula. Mantel tests for each indicated p <0.05 for all populations except Tamar West, and was incalculable for North East due a lack of coordinate data for these samples.


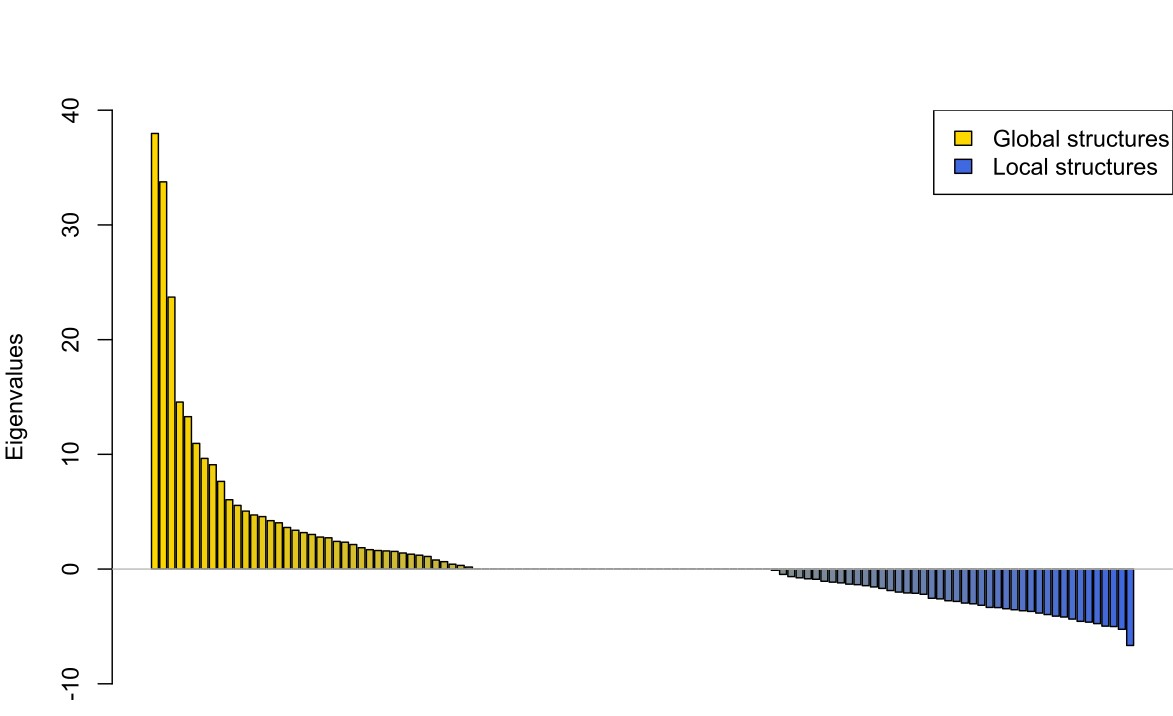

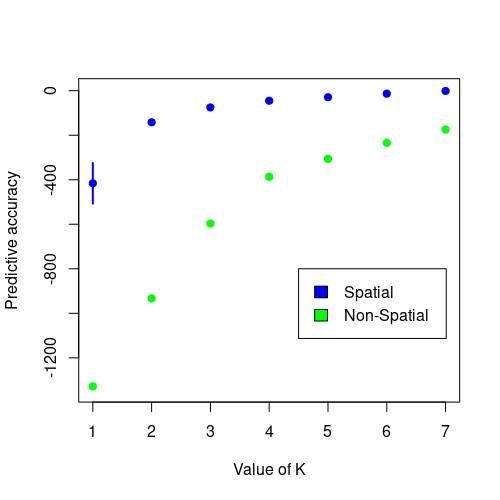

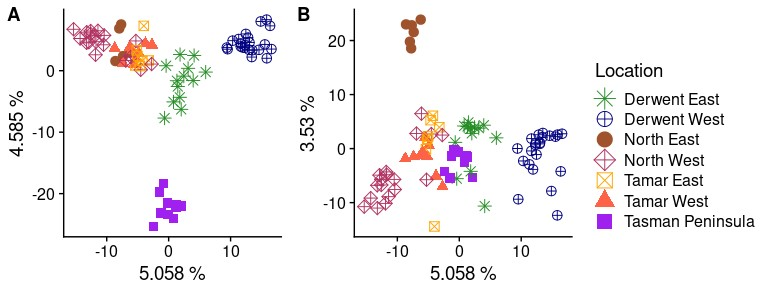

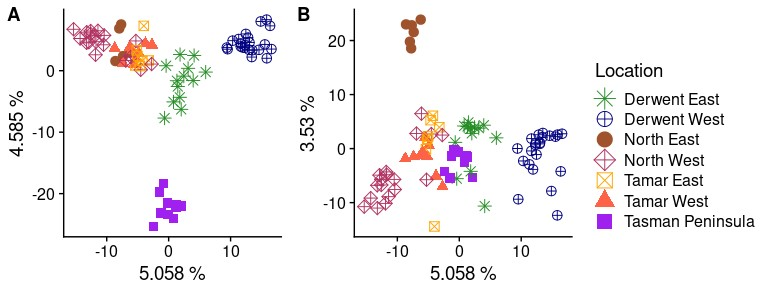


Supplementary figure 4: **A)** Relative levels of predictive accuracy of various values of K for ConStruct spatial and non-spatial assessment of isolation by distance. The spatial model accounts for decay of genotype similarity over space, while the non-spatial model does not. Error-bars represent 95% confidence intervals (most are non-visible). **B)** sPCA barplot of eigenvectors of spatially autocorrelated genotype similarity, predictive of global and local structures. Strongly positive components are suggestive of global structures, while strongly negative components are suggestive of local structures.
